# Blood Pressure and Heart Rate Variability and the Impact on Pregnancy Outcomes: A Systematic Review

**DOI:** 10.1161/JAHA.123.032636

**Published:** 2024-02-27

**Authors:** Milly G. Wilson, Jeffrey N. Bone, Hiten D. Mistry, Laura J. Slade, Joel Singer, Peter von Dadelszen, Laura A. Magee

**Affiliations:** ^1^ Department of Women and Children’s Health School of Life Course and Population Health Sciences, Faculty of Medicine, King’s College London UK; ^2^ British Columbia Children’s Hospital Research Institute, University of British Columbia Vancouver Canada; ^3^ Department of Obstetrics and Gynaecology University of British Columbia Vancouver Canada; ^4^ Robinson Research Institute, The University of Adelaide South Australia Australia; ^5^ Department of Obstetrics and Gynaecology Women’s and Children’s Hospital Adelaide Australia; ^6^ School of Population and Public Health University of British Columbia Vancouver Canada

**Keywords:** blood pressure variability, fetal growth, hypertension, pregnancy, High Blood Pressure, Hypertension, Preeclampsia, Blood Pressure, Meta Analysis

## Abstract

**Background:**

Long‐term (visit‐to‐visit) blood pressure variability (BPV) and heart rate variability (HRV) outside pregnancy are associated with adverse cardiovascular outcomes. Given the limitations of relying solely on blood pressure level to identify pregnancies at risk, long‐term (visit‐to‐visit) BPV or HRV may provide additional diagnostic/prognostic counsel. To address this, we conducted a systematic review to examine the association between long‐term BPV and HRV in pregnancy and adverse maternal and perinatal outcomes.

**Methods and Results:**

Databases were searched from inception to May 2023 for studies including pregnant women, with sufficient blood pressure or heart rate measurements to calculate any chosen measure of BPV or HRV. Studies were excluded that reported short‐term, not long‐term, variability. Adjusted odds ratios were extracted. Eight studies (138 949 pregnancies) reporting BPV met our inclusion criteria; no study reported HRV and its association with pregnancy outcomes. BPV appeared to be higher in women with hypertension and preeclampsia specifically, compared with unselected pregnancy cohorts. Greater BPV was associated with significantly more adverse pregnancy outcomes, particularly maternal (gestational hypertension [odds ratio range, 1.40–2.15], severe hypertension [1.40–2.20]), and fetal growth (small‐for‐gestational‐age infants [1.12–1.32] or low birth weight [1.18–1.39]). These associations were independent of mean blood pressure level. In women with hypertension, there were stronger associations with maternal outcomes but no consistent pattern for perinatal outcomes.

**Conclusions:**

Future work should aim to confirm whether BPV could be useful for risk stratification prospectively in pregnancy, and should determine the optimal management path for those women identified at increased risk of adverse outcomes.

**Nonstandard Abbreviations and Acronyms**
BPV: blood pressure variability
CHIPS: Control of Hypertension in Pregnancy Study
CLIP: Community‐Level Interventions for Pre‐eclampsia
HR: heart rate
HRV: heart rate variability

**Clinical Perspective**

**What Is New?**

- This is the first systematic review to explore the association between visit‐to‐visit blood pressure variability and adverse pregnancy outcomes.
- Higher blood pressure variability is associated with adverse maternal and perinatal outcomes, independently of mean blood pressure.
- The link between higher blood pressure variability and maternal outcomes appeared to be stronger in women with hypertension specifically.

**What Are the Clinical Implications?**

- If blood pressure variability is confirmed as useful for risk stratification prospectively, it could provide important prognostic information and improve outcomes in pregnancy hypertension.

Visit‐to‐visit blood pressure variability (BPV) refers to dynamic fluctuations in blood pressure (BP) across days, months, and years. These oscillations in BP reflect the integrated impact of behavior (eg, lifestyle), environment (eg, ambient noise), and biology (eg, decreased arterial elasticity and intrinsic arterial and cardiopulmonary reflexes).^1^ Outside pregnancy, higher visit‐to‐visit BPV is associated with greater cardiovascular risk.^2^

Given the limitations of relying solely on BP level to identify women and babies at increased risk of complications,^3^ there is a need to explore additional elements of BP to improve risk stratification. As such, attention has turned to exploring whether higher BPV is associated with adverse outcomes in pregnancy, a population with unique cardiovascular physiology. It is possible that rapid rises and falls in BP during pregnancy reflect an inability to maintain hemodynamic homeostasis and may be associated with progression to the hypertensive disorders of pregnancy and other adverse pregnancy outcomes. If so, BPV may provide important prognostic information to BP level, particularly when BP has not yet reached the threshold for diagnosis of hypertension (ie, 140/90 mm Hg).

Also, visit‐to‐visit heart rate variability (HRV) is associated with higher cardiovascular risk outside pregnancy.^4^ Heart rate (HR) is routinely measured by automated BP measurement devices in pregnancy but is poorly recorded in maternity records, and it is not known whether HR is related to adverse pregnancy outcomes.

The objective of this study was to synthesize, in the form of a systematic review, the available published literature on the association, if any, between visit‐to‐visit BPV or visit‐to‐visit HRV and the risk of maternal and perinatal adverse outcomes.

## Methods

The authors declare that all supporting data are available within the article (and its online supplementary files). Institutional review board approval was not deemed necessary, as the study did not involve individual patient data or primary data collection. Obtaining informed consent from study participants was not applicable.

### Study Design

We conducted a systematic review to evaluate the association between adverse pregnancy outcomes and each of visit‐to‐visit BPV and HRV. The protocol for this review was registered on the International Prospective Register of Systematic Reviews (reference CRD42022288772). We followed the guidelines for systematic reviews outlined by the Preferred Reporting Items for Systematic Reviews and Meta‐Analyses statement (PRISMA) (Table S1).

### Search Strategy

The databases MEDLINE and EMBASE (via Ovid), PubMed, and Web of Science were searched for eligible articles from inception in February 2022, with active monthly monitoring until May 2023. Search strategies were tailored to each database, using Medical Subject Headings where applicable, to increase sensitivity to suitable studies. Searches were limited to English language and human subjects where these options were available. A detailed search strategy is supplied in Table S2.

### Eligibility Criteria

Eligible studies were those that (1) included only pregnant women, with (2) the calculation of either visit‐to‐visit BPV or visit‐to‐visit HRV (long‐term, between antenatal clinic visits throughout pregnancy), by any chosen measure of BPV or HRV (eg, SD, which reflects dispersion of measurements around mean BP; average real variability (ARV), the average of absolute successive differences of all BP values, reflecting changes over short time intervals; coefficient of variation (CV), the ratio of the SD to the mean of BP), presented either as a continuous variable or in quantiles, and (3) recorded the association between either BPV or HRV and pregnancy outcomes. We excluded studies that either reported very short‐term (seconds or minutes, beat‐to‐beat), short‐term (within 24 hours) or midterm (day‐to‐day) BPV. We excluded studies that did not report relevant pregnancy outcomes. We excluded studies that reported HRV using electrocardiography (as beat‐to‐beat HRV).

Using Rayyan software,^5^ at least 2 of 4 authors (M.W., H.M., L.M., and P.v.D.) independently screened all titles and abstracts and then full texts against the eligibility criteria. Disagreements were resolved by consensus.

### Data Extraction and Quality Assessment

For each included paper, 2 authors independently abstracted relevant data using a prespecified abstraction table that included study characteristics (eg, design, setting, population, inclusion/exclusion criteria, year of publication), participant characteristics (eg, age, body mass index, ethnicity, smoking status, parity) and their BP (eg, number of BP and HR measurements, mean BP, and BPV and HRV), pregnancy outcomes, and minimally and maximally adjusted measures of association with adverse pregnancy outcomes, including 95% CIs. Any discrepancies were resolved by discussion, with a third reviewer if necessary.

The main outcomes are defined in Table 1: gestational hypertension, preeclampsia, severe hypertension, serious maternal complications, perinatal death, preterm birth, small‐for‐gestational‐age (SGA) infants, and neonatal intensive care unit admission. Secondary maternal outcomes were maternal death, admission to intensive care, placental abruption, cesarean section, and postpartum hemorrhage. Other severe maternal morbidities that are core outcomes in pregnancy hypertension were not included individually, as they occur infrequently and were part of the composite severe maternal morbidity outcome.^6^ Secondary neonatal outcomes were 5‐minute Apgar score, respiratory support, and neonatal seizures. Not examined other than for serious maternal complications was intubation or ventilation (other than for childbirth). The core outcomes of low platelets and elevated liver enzymes were included in the definition of preeclampsia. We extracted the sensitivity analyses of included studies, such as the number of BP measurements used to calculate BPV, the additional discriminative ability of BPV over mean BP, analyses stratified by common confounders, and exploration of reverse causality.

**Table 1:** Maternal and Perinatal Outcomes*

Maternal outcomes	Perinatal outcomes
Primary outcomes	
Gestational hypertension (BP ≥140/90 mm Hg without proteinuria or other clinical features suggestive of preeclampsia, diagnosed at ≥20 weeks' gestation)	Small‐for‐gestational‐age infants (birth weight <10th or 3rd centile)
Preeclampsia (as defined and documented)	Admission to neonatal intensive care unit/special care baby unit
Severe hypertension (BP ≥160/110 mm Hg)	Preterm delivery (either <37 or <34 weeks' gestation)
Serious maternal complications (≥1 of the following: stroke, cortical blindness, retinal detachment, pulmonary edema, acute kidney injury, liver capsule hematoma or rupture)	Fetal or neonatal death (miscarriage, stillbirth, or neonatal death)
Secondary Outcomes	
Maternal death	Apgar score 5 min after birth
Admission to intensive care unit	Respiratory support
Disseminated intravascular coagulation	Neonatal seizures
Hemolysis, elevated liver enzymes, low platelet count	
Eclampsia	
Placental abruption	
Cesarean delivery	
Postpartum hemorrhage	

* When not specified, outcomes were defined as per the author.

The QUADAS‐2 (Quality Assessment of Diagnostic Accuracy Studies) tool for assessment of the quality of primary diagnostic accuracy studies was used to assess risk of bias and applicability. QUADAS‐2 has 4 sections: patient selection, index test (ie, BPV or HRV in this study), reference standard (BP level) and flow and timing of patient inclusion and follow‐up. Study bias was classified as *high*, *low*, or *unclear* for each section. Each paper received 2 bias ratings by separate reviewers. Discussion with a third reviewer resolved discrepancies.

The results of sensitivity analyses were summarized narratively. BPV was summarized graphically and tabulated by hypertensive disorders of pregnancy status. When BPV was presented in quartiles, the impact on outcomes of the fourth (highest) versus first (lowest) quartiles was presented.

## Results

### Literature Search

**Figure 1:**
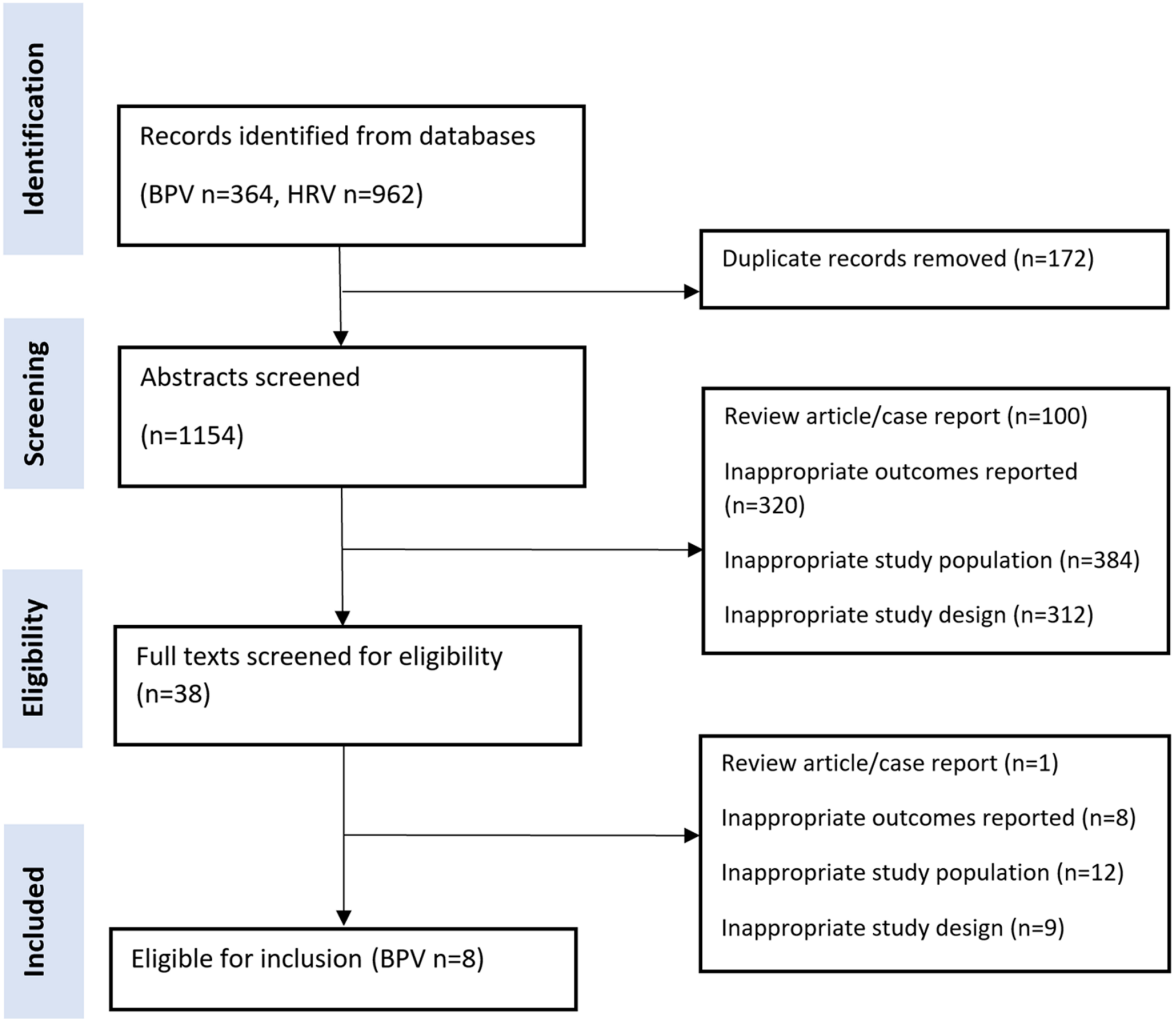
Preferred Reporting Items for Systematic Reviews and Meta‐Analyses flow diagram for study inclusion and exclusion. BPV indicates blood pressure variability; and HRV, heart rate variability.

As summarized in Figure 1, our search yielded 364 records for BPV and 962 records for HRV. After excluding duplicates (N=172) and ineligible studies at either title/abstract screening (N=100) or full‐text review (N=30), 8 studies reporting on BPV in pregnancy met our inclusion criteria.^7^, ^8^, ^9^, ^10^, ^11^, ^12^, ^13^, ^14^ No eligible study reported on HRV and its association with pregnancy outcomes.

### Study Characteristics

Detailed characteristics of the 8 included studies and their participants are presented in Tables S3 through S7.

Included articles were published between 2019 and 2022 (Table S3). Six studies were from higher‐ to middle‐income countries,^7^, ^8^, ^10^, ^12^, ^13^, ^14^ 1 was multicountry from 15 countries of mixed economic levels,^9^ and 1 was from 3 low‐income countries.^11^ The studies reported on 138 949 participants (median per study, 9433; range, 140–52 891). Two studies were cross sectional,^12^, ^13^ and 6 were retrospective in design,^7^, ^8^, ^9^, ^10^, ^11^, ^14^ including 2 secondary analyses of randomized trials.^9^, ^11^ Studies included women either throughout pregnancy,^9^, ^11^, ^14^ or focused on the second half of pregnancy^7^, ^8^, ^10^, ^12^, ^13^ (termed *unselected pregnancy* cohorts). Four studies excluded patients with high BP (ie, ≥140/90 mm Hg) or proteinuria present before 20 weeks' gestation^7^, ^8^, ^10^, ^14^ (termed *normotensive* cohorts), and 2 were specifically restricted to women with hypertension (termed *hypertensive* cohorts), as secondary analyses of randomized trials.^9^, ^11^

BPV measurement characteristics are presented in Table S4. BPV was evaluated by 3 metrics: SD (all studies), CV (N=6^7^, ^8^, ^10^, ^12^, ^13^, ^14^), and ARV (N=3^9^, ^11^, ^13^). All studies evaluated systolic BPV, all but 2 evaluated diastolic BPV,^7^, ^12^ and 1 also evaluated mean arterial pressure.^10^ Most studies measured BP using a mercury sphygmomanometer, with women in a seated position, following 5 minutes' rest. To calculate BPV, studies required a minimum of 2,^9^, ^11^ 3,^7^, ^8^, ^10^ or 6^14^ BP measurements (or the minimum was not specified),^12^, ^13^ at office/clinic visits specifically in 5 studies.^7^, ^9^, ^12^, ^13^, ^14^ Also, BPV was presented as a continuous variable (N=6^7^, ^8^, ^9^, ^11^, ^12^, ^13^) or in quartiles (N=2^10^, ^14^), where analyses were presented as second, third, or fourth quartile versus the first.

Six studies reported the association between BPV and pregnancy outcomes (Table S3); the 2 additional studies presented only values of BPV stratified by pregnancy hypertension status.^12^, ^13^ Outcomes were variably reported by the 6 relevant studies, with no outcome reported by all studies: gestational hypertension and preeclampsia (N=6^7^, ^8^, ^9^, ^11^, ^12^, ^13^), preterm birth at <37 weeks' gestation (N=4^9^, ^10^, ^11^, ^12^, ^13^), SGA infants (N=3^9^, ^10^, ^14^), low birth weight (N=1^10^), stillbirth (N=2^9^, ^11^), early and late neonatal death (N=1^11^), or a composite of maternal outcomes (N=2^7^, ^11^), perinatal outcomes (N=2^9^, ^11^), or both (N=3^7^, ^9^, ^11^). All of these studies adjusted BPV for BP level, and most reported adjustment for other covariates, usually maternal age, body mass index, parity, and pregnancy weight gain.

Risk of bias measured by the QUADAS‐2 tool is reported in Table S5. All studies were at low risk of bias, based on low risk of applicability concerns for the index test, reference standard, and flow and timing. However, the risk of bias for patient selection was high for 4 studies, due to exclusion of women with chronic hypertension or proteinuria at baseline.^7^, ^8^, ^10^, ^14^

Characteristics of participants are presented in Table S6. Women were ≈30 years of age with an early pregnancy body mass index of ≈21.5 kg/m^2^ in unselected pregnancy cohorts, and 29.9 kg/m^2^ for studies including participants with hypertension. Only 1 study described ethnicity, but 4 studies were from East Asian countries.^7^, ^8^, ^10^, ^14^ No studies reported on smoking status or alcohol consumption during pregnancy. Only 1 study included multiple births,^7^ although no study specifically excluded them. Three studies reported on antihypertensive medication use among women with hypertension.^9^, ^12^, ^13^ On average, women gave birth at term.

### BPV and Pregnancy Outcomes

**Figure 2:**
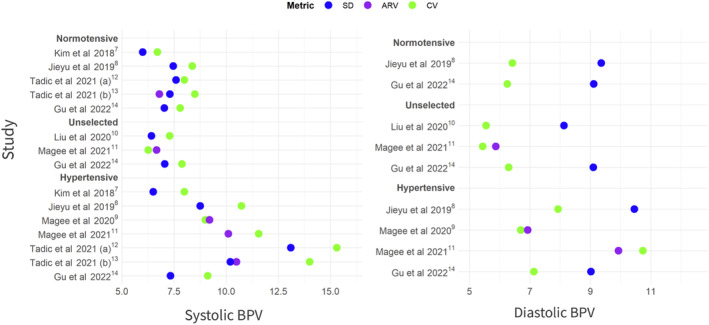
Mean BPV stratified by participant group. Individual points represent mean values from individual studies. ARV indicates average real variability; BPV, blood pressure variability; and CV, coefficient of variation.

BPV values are presented descriptively in Figure 2, with numeric details presented in Table S7. BPV appeared higher in hypertensive cohorts and those with preeclampsia specifically (Table S7), compared with normotensive or unselected pregnancy cohorts, among whom BPV was lowest. This pattern was true for systolic and diastolic BPV measures, although less clearly so with SD as a BPV measure for diastolic BP.

In unselected pregnancy and normotensive cohorts combined, across BPV measures, there was a general pattern of association between greater BPV and significantly more adverse pregnancy outcomes, particularly maternal (Table 2). Associations were particularly strong for gestational hypertension (odds ratio, 1.40–2.15) and severe hypertension (odds ratio, 1.40–2.20) for the mother, and for the baby, growth (odds ratio for SGA infants, 1.12–1.32, and for low birth weight, 1.18–1.39).

**Table 2:** Association Between BPV and Maternal and Perinatal Outcomes (Odds Ratio and 95% CI)*

All subjects	Systolic BP			Diastolic BP		
	SD	ARV	CV	SD	ARV	CV
Maternal outcomes						
Gestational hypertension	1.49 (1.44–1.54)	1.40 (1.34–1.47)	1.62 (1.56–1.68)	1.57 (1.51–1.63)	1.65 (1.57–1.73)	1.40 (1.36–1.44)
	1.78 (1.70–1.88)			2.15 (2.01–2.27)		
Preeclampsia	1.13 (1.06–1.19)	‐	1.14 (1.06–1.21)	1.05 (0.97–1.14)	‐	1.03 (0.97–1.09)
Severe hypertension	2.20 (1.98–2.46)	‐	‐	1.98 (1.79–2.20)	1.40 (1.26–1.55)	‐
Maternal composite	1.08 (1.03–1.14)	‐	‐	1.08 (1.02–1.13)	1.05 (1.00–1.11)	‐
Maternal death	1.23 (0.96–1.59)	1.19 (0.94–1.50)	‐	1.39 (1.14–1.70)	1.35 (1.14–1.60)	‐
Maternal morbidity	1.08 (1.02–1.13)	1.04 (0.99–1.09)	‐	1.08 (1.02–1.13)	1.05 (1.00–1.10)	‐
Perinatal outcomes						
Preterm birth	1.01 (0.98–1.06)	1.07 (1.03–1.11)	‐	0.99 (0.95–1.03)	1.05 (1.02–1.10)	‐
BPV fourth vs first quartile	1.01 (0.89–1.14)	‐	1.00 (0.89–1.13)	1.11 (0.98–1.25)	‐	1.16 (1.03–1.31)
	0.96 (0.86–1.08)		0.97 (0.86–1.09)	0.95 (0.85–1.07)		0.95 (0.84–1.08)
SGA infants	‐	‐	‐	‐	‐	‐
BPV fourth vs first quartile	1.12 (1.03–1.23)	‐	1.12 (1.02–1.23)	1.15 (1.06–1.26)	‐	1.14 (1.05–1.25)
	1.35 (1.16–1.58)		1.26 (1.08–1.47)	1.32 (1.14–1.54)		1.38 (1.17–1.61)
LBW	‐	‐	‐	‐	‐	‐
BPV fourth vs first quartile	1.31 (1.11–1.53)	‐	1.22 (1.04–1.44)	1.28 (1.09–1.50)	‐	1.39 (1.18–1.63)
Fetal distress	‐	‐	‐	‐	‐	‐
BPV fourth vs first quartile	1.05 (0.98–1.12)	‐	1.05 (0.98–1.12)	1.19 (1.11–1.27)	‐	1.22 (1.14–1.30)
Perinatal composite	1.08 (1.04–1.13)	1.06 (1.02–1.11)	‐	1.05 (1.01–1.09)	1.05 (1.01–1.10)	‐
Stillbirth	1.12 (1.04–1.20)	1.12 (1.05–1.20)	‐	1.10 (1.03–1.18)	1.12 (1.05–1.20)	‐
Early NND	0.98 (0.90–1.07)	1.00 (0.92–1.09)	‐	0.98 (0.90–1.07)	1.00 (0.92–1.09)	‐
Late NND	1.11 (0.95–1.30)	1.07 (0.91–1.25)	‐	1.03 (0.88–1.20)	1.03 (0.89–1.20)	‐
Neonatal morbidity	1.09 (1.04–1.15)	1.05 (1.00–1.10)	‐	1.05 (1.00–1.10)	1.02 (0.97–1.08)	‐
Apgar score	‐	‐	‐	‐	‐	‐
BPV fourth vs first quartile	0.66 (0.33–1.31)	‐	0.57 (0.28–1.14)	0.84 (0.44–1.62)	‐	0.80 (0.39–1.66)
Pregnancy composite	1.16 (1.04–1.30)	‐	1.13 (1.02–1.25)	1.07 (1.03–1.11)	1.06 (1.02–1.09)	‐
	1.10 (1.06–1.14)					

Subjects with hypertension	Systolic BP			Diastolic BP		
	SD	ARV	CV	SD	ARV	CV
Maternal outcomes						
Gestational hypertension	‐	‐	‐	‐	‐	‐
Preeclampsia	1.23 (1.04–1.44)	1.29 (1.10–1.53)	‐	1.33 (1.13–1.56)	1.32 (1.11–1.57)	‐
Severe hypertension	1.80 (1.47–2.20)	1.60 (1.32–1.94)	‐	1.42 (1.17–1.71)	1.29 (1.07–1.57)	‐
Maternal composite	1.27 (0.91–1.78)	1.25 (0.92–1.69)	‐	1.25 (0.88–1.75)	1.17 (0.86–1.61)	‐
	1.26 (1.02–1.56)	1.02 (0.82–1.28)		1.02 (0.82–1.28)	1.20 (0.97–1.48)	
Maternal death	2.07 (1.14–3.76)	1.69 (1.05–2.71)	‐	1.99 (0.74–5.37)	1.97 (0.94–4.12)	‐
Maternal morbidity	1.23 (0.98–1.53)	0.98 (0.77–1.25)	‐	1.40 (1.12–1.76)	1.21 (0.97–1.50)	‐
Perinatal outcomes						
Preterm birth	1.18 (1.00–1.39)	1.21 (1.03–1.43)	‐	0.90 (0.76–1.07)	0.95 (0.81–1.12)	‐
SGA infants	1.03 (0.86–1.23)	1.00 (0.85–1.19)	‐	0.84 (0.70–1.01)	0.88 (0.73–1.06)	‐
Perinatal composite	1.05 (0.89–1.24)	1.17 (0.99–1.38)	‐	0.85 (0.72–1.01)	0.93 (0.78–1.10)	‐
	1.19 (0.93–1.52)	1.13 (0.95–1.36)		1.09 (0.91–1.31)	1.00 (0.82–1.21)	
Stillbirth	1.23 (0.79–1.92)	1.48 (1.03–2.13)	‐	1.08 (0.68–1.72)	1.29 (0.91–1.83)	‐
	1.08 (0.76–1.52)	1.05 (0.83–1.35)		1.05 (0.80–1.36)	0.95 (0.71–1.26)	
Early NND	1.32 (0.71–2.42)	1.03 (0.74–1.44)	‐	1.13 (0.80–1.61)	1.03 (0.71–1.50)	‐
Late NND	1.24 (0.98–1.56)	1.39 (0.85–2.25)	‐	0.80 (0.40–1.62)	0.99 (0.49–1.97)	‐
Neonatal morbidity	1.03 (0.87–1.22)	1.13 (0.96–1.33)	‐	0.84 (0.70–1.00)	0.89 (0.75–1.06)	‐
	1.21 (0.89–1.66)	1.29 (1.02–1.63)		1.14 (0.89–1.45)	1.13 (0.89–1.45)	
Pregnancy composite	1.32 (1.12–1.56)	1.14 (0.96–1.35)	‐	1.21 (1.03–1.43)	1.09 (0.92–1.29)	‐

ARV indicates average real variability; BP, blood pressure; BPV, blood pressure variability; CV, coefficient of variation; LBW, low birth weight; NND, neonatal death; and SGA, small‐for‐gestational age.

(−) indicates that no data were available. Individual cells' effect estimates reflect results from 1 single study.

* The odds ratios and 95% CIs reflect the relationship between BPV as a continuous variable and outcomes as presented by studies, or as the fourth quartile of BPV versus the first (as presented by 2 studies).

In hypertensive cohorts, a similar pattern of effect was seen (as reflected by yellow shading in Table 2), with the strength of association with adverse maternal outcomes as strong or stronger, particularly for maternal death (odds ratio, 1.69–2.07). Importantly, there was little evidence of an association of higher BPV with adverse perinatal outcomes and no protective effect evident.

For detailed outcomes by study, see Tables S8 (maternal) and S9 (perinatal) for BPV as a continuous variable, and Table S10 for BPV assessed in quartiles.

### Sensitivity Analysis

To examine potential *reverse causality*, secondary analyses of the CHIPS (Control of Hypertension in Pregnancy Study)^9^ and the CLIP (Community‐Level Interventions for Pre‐eclampsia)^11^ trials excluded BP values 7, 14, 21, and 28 days before the outcomes of interest. In CHIPS (a hypertensive cohort), this attenuated the relationship between higher BPV and adverse maternal outcomes, but the apparent association between more diastolic BPV and fewer adverse perinatal outcomes increased.^9^ In CLIP (an unselected pregnancy cohort), while there was no longer an association between systolic BPV and outcomes, the association between diastolic BPV and maternal death and stillbirth remained, and the strength of association between BPV and adverse perinatal outcomes remained similar.^11^ Adjusting for the last BP before birth in each of the CHIPS and CLIP trials did not substantially change results.^9^, ^11^

To examine the impact of examining BPV–outcome associations in women with at least 2 (rather than 3) BP records, Liu et al produced similar results.^10^ In additional analyses stratified by common confounders (of maternal age, body mass index, hypertensive disorders of pregnancy, gestational diabetes, parity, term delivery, and infant sex), associations between diastolic BPV and SGA were similar to the main results.^10^

In one study, adding systolic BP CV to established hypertensive disorders of pregnancy risk factors improved the identification of participants at risk of gestational hypertension, based on an increase in the concordance statistic from 0.87 (95% CI, 0.86–0.88) to 0.92 (95% CI, 0.91–0.93).^8^

## Discussion

### Principal Findings

In this systematic review of 8 studies and almost 140 000 women, higher BPV in pregnancy was associated with a higher risk of adverse maternal outcomes. BPV was most commonly assessed by SD and the association adjusted for both BP level and established risk factors for adverse pregnancy outcomes.

Among unselected pregnancies, higher BPV was associated with adverse maternal outcomes, most notably gestational hypertension, and severe hypertension, as well as adverse perinatal outcomes, particularly occurrence of smaller babies (SGA and low birth weight) and stillbirth.

Among hypertensive pregnancies specifically, the link between higher BPV and development of adverse maternal outcomes appeared to be stronger than in unselected pregnancies. However, there was no consistent association between higher BPV and more adverse perinatal outcomes, and certainly no evidence that higher BPV was protective for the baby.

Of note, no studies were identified that evaluated the relationship between visit‐to‐visit HRV and pregnancy outcomes.

### Comparison With the Literature and Interpretation

To the best of our knowledge, this is the first systematic review of BPV and its association with pregnancy outcomes.

Our findings of the association between higher BPV, adjusted for BP level, and adverse outcomes in pregnancy is consistent with literature associating higher BPV with adverse health outcomes outside pregnancy. In a review of 19 observational cohort studies and 17 clinical trial cohorts, higher systolic visit‐to‐visit BPV in office BP measurements, independent of BP level, was associated with higher all‐cause and cardiovascular disease death, as well as more cardiovascular disease events, including stroke.^2^ Underscoring the growing recognition of the significance of BPV in managing cardiovascular risk,^15^ systolic BP SD has been included in the QRISK cardiovascular risk calculators for 10‐year (QRISK3–2018, https://qrisk.org) and lifetime (QRISK3‐lifetime, https://qrisk.org/lifetime) time frames.

If BPV were used to identify pregnancies at increased risk, this would be an important advance. It appears that clinical advances will not come from lowering the threshold for hypertension in pregnancy.^3^, ^16^ In 2 systematic reviews, no BP threshold (even one above a *normal* BP of <120/80 mm Hg by American College of Cardiology/American Heart Association criteria) could reassure about development of adverse outcomes, and only BP ≥140/90 mm Hg (ie, the current threshold for hypertension) increased the likelihood of adverse pregnancy outcomes: preeclampsia when BP was ≥140/90 mm Hg occurred at <20 weeks' gestation (23 studies, 734 377 participants),^16^ and preeclampsia, stroke, maternal death, and stillbirth when BP was ≥140/90 mm Hg occurred in the second half of pregnancy (12 studies, 251 172 participants).^3^

All included studies retrospectively evaluated the relationship between pregnancy outcome and BPV over the course of pregnancy. However, to optimize usefulness in clinical care, BPV should be of prognostic value when used prospectively. It will be critical to determine the optimal method for assessing BPV (ie, SD, ARV, or CV), how to update BPV calculation, based on accumulating numbers of BP measurements taken with advancing gestational age, the impact of normal BP changes (eg, the midtrimester dip) on BPV by incorporating the trajectory of BP, whether to adjust for important covariates or effect modifiers of the BPV–outcome relationship, including antihypertensive therapy, the mechanism by which BPV is related to outcomes, maternal and perinatal, and whether the relationship is mediated through physiological or iatrogenic effects (eg, different antihypertensive agents). In a meta‐analysis (nonpregnant subjects), calcium channel blockers were shown to decrease BPV, whereas angiotensin receptor blockers and β blockers increase it.^17^ Whether this is true in pregnancy should be investigated, with particular attention paid to oral labetalol, an α and β blocker that is used extensively in pregnancy (but not outside) for treatment of hypertension.

How should those identified as being at increased risk be managed? Likely through enhanced surveillance, as there is no evidence to support treatment of increased BPV outside pregnancy. Exploring whether additional value could be added by including visit‐to‐visit HRV during pregnancy would be worthwhile, particularly as HR is recorded by automated BP machines automatically; a prior systematic review of beat‐to‐beat HR variability supported the hypothesis that sympathetic overdrive in the hypertensive disorders of pregnancy is associated with parasympathetic withdrawal, but there was large between‐study heterogeneity.^18^

### Strengths and Limitations

The strengths of this study are inclusion of a diverse range of women from multiple countries at varying stages of development, with representation from low‐ and middle‐income countries where the burden occurs of adverse outcomes related to pregnancy hypertension. Also, most studies reported standardized BP measurement.

This review has limitations. First, a significant constraint is the quality and quantity of evidence, with all outcomes other than preterm birth reported by only 1 or 2 studies, precluding meta‐analysis; we suggest this work be updated in coming years as a living review. Second, multiple comparisons were made, and we acknowledge the possibility of type I errors. Third, the number of visits and the time interval between them may have introduced additional influences on the association between BPV and outcomes. Although there has been criticism of using a minimum of 2 BP measurements to calculate SD as a BPV measure,^15^ Liu et al found no impact on results, compared with use of at least 3 BP measurements.^10^ Fourth, there is concern that BPV variability simply reflects high BP level; however, all studies included in this review adjusted for BP level. Fifth, visit‐to‐visit BPV may also reflect measurement methodologies, but this is reflective of real‐world practice. Sixth, most data relate to unselected pregnancies, although this review provides some evidence of the association between BPV and outcomes in hypertensive pregnancy, primarily severe hypertension, or preeclampsia. Nevertheless, the impact of BPV on outcomes is unknown in women with proteinuria at booking or multiple gestations. Seventh, antihypertensive medication prescription was not reported, precluding study of the influence of medication adherence patterns, dosage, and duration on BPV and adverse outcomes; however, when treatment BP goal was accounted for in the CHIPS trial,^9^ the BPV–outcome relationship was evident. Finally, the ethnic composition of the study populations was neither reported nor adjusted for in the majority of included studies.

### Conclusions

This systematic review has confirmed an association between visit‐to‐visit BPV in pregnancy and adverse outcomes, independently of mean BP levels, in normotensive subjects and subjects with hypertension. If confirmed as useful prospectively for risk stratification, BPV could provide important prognostic information and improve outcomes in pregnancy hypertension, a leading cause of maternal and fetal/newborn death and morbidity worldwide.

## Sources of Funding

M. Wilson was funded by the King's College London Centre for Doctoral Training in Data‐Driven Health (ST12512). The PRECISE (Pregnancy Care Integrating Translational Science, Everywhere) Network is funded by the UK Research and Innovation Grand Challenges Research Fund GROW Award scheme (MR/P027938/1). For the purpose of open access, the author has applied a Creative Commons Attribution license to any author accepted manuscript version arising from this submission. This work was supported by funding from a UK Research and Innovation Global Challenges Research Fund GROW award (MR/P027938/1).

## Disclosures

None.

## Supporting information

Tables S1–S10
