# Supplementary material for: Blood Pressure and Heart Rate Variability and the Impact on Pregnancy Outcomes: A Systematic Review
Source: J Am Heart Assoc. 2024 Feb 27;13(5):e032636. doi: 10.1161/JAHA.123.032636 (PMC10944029; doi:10.1161/JAHA.123.032636)
Supplement: Supplementary file 1 — Tables S1–S10 [file JAH3-13-e032636-s001.pdf]

# SUPPLEMENTAL MATERIAL

**Table S1.** PRISMA Item Checklist

| Section and topic | Item # | Checklist item | Location |
|---|---|---|---|
| **Title** | | | |
| Title | 1 | Identify the report as a systematic review. | 1 |
| **Abstract** | | | |
| Abstract | 2 | Provide a structured summary including, as applicable: background; objectives; data sources; study eligibility criteria, participants, and interventions; study appraisal and synthesis methods; results; limitations; conclusions and implications of key findings; systematic review registration number. | 2 |
| **Introduction** | | | |
| Rationale | 3 | Describe the rationale for the review in the context of existing knowledge. | 3 |
| Objectives | 4 | Provide an explicit statement of the objective(s) or question(s) the review addresses. | 3 |
| **Methods** | | | |
| Eligibility criteria | 5 | Specify the inclusion and exclusion criteria for the review and how studies were grouped for the syntheses. | 3,4 |
| Information sources | 6 | Specify all databases, registers, websites, organisations, reference lists and other sources searched or consulted to identify studies. Specify the date when each source was last searched or consulted. | 3 |
| Search strategy | 7 | Present the full search strategies for all databases, registers, and websites, including any filters and limits used. | Table S2 |
| Selection process | 8 | Specify the methods used to decide whether a study met the inclusion criteria of the review, including how many reviewers screened each record and each report retrieved, whether they worked independently, and if applicable, details of automation tools used in the process. | 4 |
| Data collection process | 9 | Specify the methods used to collect data from reports, including how many reviewers collected data from each report, whether they worked independently, any processes for obtaining or confirming data from study investigators, and if applicable, details of automation tools used in the process. | 4 |
| Data items | 10a | List and define all outcomes for which data were sought. Specify whether all results that were compatible with each outcome domain in each study were sought (e.g. for all measures, time points, analyses), and if not, the methods used to decide which results to collect. | Table 1 |
| | 10b | List and define all other variables for which data were sought (e.g. participant and intervention characteristics, funding sources). Describe any assumptions made about any missing or unclear information. | 4 |

| | | | |
|---|---|---|---|
| Study risk of bias assessment | 11 | Specify the methods used to assess risk of bias in the included studies, including details of the tool(s) used, how many reviewers assessed each study and whether they worked independently, and if applicable, details of automation tools used in the process. | 4 |
| Effect measures | 12 | Specify for each outcome the effect measure(s) (e.g. risk ratio, mean difference) used in the synthesis or presentation of results. | 4 |
| Synthesis methods | 13a | Describe the processes used to decide which studies were eligible for each synthesis (e.g. tabulating the study intervention characteristics and comparing against the planned groups for each synthesis (item #5)). | 3,4 |
| | 13b | Describe any methods required to prepare the data for presentation or synthesis, such as handling of missing summary statistics, or data conversions. | 4 |
| | 13c | Describe any methods used to tabulate or visually display results of individual studies and syntheses. | 3,4 |
| | 13d | Describe any methods used to synthesise results and provide a rationale for the choice(s). If meta-analysis was performed, describe the model(s), method(s) to identify the presence and extent of statistical heterogeneity, and software package(s) used. | 4 |
| | 13e | Describe any methods used to explore possible causes of heterogeneity among study results (e.g. subgroup analysis, meta-regression). | 4 |
| | 13f | Describe any sensitivity analyses conducted to assess robustness of the synthesised results. | 4 |
| Reporting bias assessment | 14 | Describe any methods used to assess risk of bias due to missing results in a synthesis (arising from reporting biases). | 4 |
| Certainty assessment | 15 | Describe any methods used to assess certainty (or confidence) in the body of evidence for an outcome. | 4 |
| **Results** | | | |
| Study selection | 16a | Describe the results of the search and selection process, from the number of records identified in the search to the number of studies included in the review, ideally using a flow diagram (see fig 1). | 5, Figure 1 |
| | 16b | Cite studies that might appear to meet the inclusion criteria, but which were excluded, and explain why they were excluded. | 5, Figure 1 |
| Study characteristics | 17 | Cite each included study and present its characteristics. | Table S3 |
| Risk of bias in studies | 18 | Present assessments of risk of bias for each included study. | Table S5 |
| Results of individual studies | 19 | For all outcomes, present, for each study: (a) summary statistics for each group (where appropriate) and (b) an effect estimate and its precision (e.g. confidence/credible interval), ideally using structured tables or plots. | Table 2, Figure 2, Table S6, Table S7, Table S8, Table S9, Table S10. |

| | | | |
|---|---|---|---|
| Results of syntheses | 20a | For each synthesis, briefly summarise the characteristics and risk of bias among contributing studies. | 5 |
| | 20b | Present results of all statistical syntheses conducted. If meta-analysis was done, present for each the summary estimate and its precision (e.g. confidence/credible interval) and measures of statistical heterogeneity. If comparing groups, describe the direction of the effect. | NA |
| | 20c | Present results of all investigations of possible causes of heterogeneity among study results. | NA |
| | 20d | Present results of all sensitivity analyses conducted to assess the robustness of the synthesised results. | NA |
| Reporting biases | 21 | Present assessments of risk of bias due to missing results (arising from reporting biases) for each synthesis assessed. | Table S5 |
| Certainty of evidence | 22 | Present assessments of certainty (or confidence) in the body of evidence for each outcome assessed. | Table S8-10 |
| **Discussion** | | | |
| Discussion | 23a | Provide a general interpretation of the results in the context of other evidence. | 7 |
| | 23b | Discuss any limitations of the evidence included in the review. | 8 |
| | 23c | Discuss any limitations of the review processes used. | 8 |
| | 23d | Discuss implications of the results for practice, policy, and future research. | 8 |
| **Other information** | | | |
| Registration and protocol | 24a | Provide registration information for the review, including register name and registration number, or state that the review was not registered. | 3 |
| | 24b | Indicate where the review protocol can be accessed, or state that a protocol was not prepared. | 3 |
| | 24c | Describe and explain any amendments to information provided at registration or in the protocol. | 3 |
| Support | 25 | Describe sources of financial or non-financial support for the review, and the role of the funders or sponsors in the review. | 2 |
| Competing interests | 26 | Declare any competing interests of review authors. | 2 |

| **Table S2**. Detailed Search Strategy | |
|---|---|
| Medline and Embase (via OVID) – Blood Pressure | |
| | (blood pressure variabilit* or blood pressure variation* or diastolic variation* or diastolic variabilit* or systolic variabilit* or systolic variation* or blood pressure oscillation* or blood pressure fluctuation* or BPV).mp. [mp=ti, ab, hw, tn, ot, dm, mf, dv, kf, fx, dq, nm, ox, px, rx, an, ui, sy] |
| | (long-term or long term or visit to visit or visit-to-visit or VVV or vvv or intervisit or between visit or between-visit or Visit-to-Visit or Visit-to-visit).mp. [mp=ti, ab, hw, tn, ot, dm, mf, dv, kf, fx, dq, nm, ox, px, rx, an, ui, sy] |
| | exp Pregnancy Outcome/ or maternal outcome*.mp. or maternal complication*.mp. or obstetric outcome*.mp. or birth outcome*.mp. or exp Pregnancy Complications/ or gestational complication*.mp. or birth complication*.mp. or exp Obstetric Labor Complications/ or fetal complication*.mp. or neonatal complication*.mp. or preterm deliver*.mp. or preterm birth*.mp. or exp Premature Birth/ or exp Premature Birth/ or exp Obstetric Labor, Premature/ or exp Stillbirth/ or exp Fetal Death/ or exp Infant, Premature/ or prematurity.mp. or intrauterine death*.mp. or exp Abortion, Spontaneous/ or miscarriage*.mp. or exp Cesarean Section/ or c section*.mp. or exp Fetal Mortality/ or neonatal mortalit*.mp. or neonatal morbidit*.mp. or fetal morbidit*.mp. or exp Infant, Premature, Diseases/ or exp Perinatal Death/ or exp Perinatal Mortality/ or exp Hypertension, Pregnancy-Induced/ or maternal hypertension.mp. or exp Pregnancy Complications, Cardiovascular/ or gestational hypertension.mp. or exp Pre-Eclampsia/ or preeclamptic.mp. or exp Eclampsia/ or eclamptic*.mp. or exp HELLP Syndrome/ or hemolysis elevated liver enzymes low platelet count.mp. or haemolysis elevated liver enzymes low platelet count.mp. or exp Disseminated Intravascular Coagulation/ or exp Abruptio Placentae/ or placental abruption.mp. or exp Postpartum Period/ or postpartum complication*.mp. or exp Intensive Care Units, Neonatal/ or NICU.mp. or exp Intensive Care, Neonatal/ or SCBU.mp. or special care baby unit*.mp. or exp Infant, Small for Gestational Age/ or SGA.mp. or Infant, Low Birth Weight/ or LBW.mp. or intrauterine growth restriction.mp. or exp Fetal Growth Retardation/ or IUGR.mp. or exp Infant, Very Low Birth Weight/ or VLBW.mp. or exp Apgar Score/ or exp Diabetes, Gestational/ or neonatal intraventricular haemorrhage*.mp. or neonatal intraventricular hemorrhage*.mp. or intraventricular haemorrhage of newborn*.mp. or intraventricular hemorrhage of newborn*.mp. or EPH gestosis.mp. or hypertension edema proteinuria gestosis.mp. or hypertension-edema-proteinuria-gestosis.mp. |
| | 1 and 2 and 3 |
| | limit 4 to English language |
| | limit 5 to humans |
| PubMed – Blood Pressure | |
| Filters: Humans, English | |
| | ((((((((blood pressure variabilit*) OR (blood pressure variation*)) OR (diastolic variation*)) OR (diastolic variabilit*)) OR (systolic variation*)) OR (systolic variabilit*)) OR (blood pressure oscillation*)) OR (blood pressure fluctuation*)) OR (BPV) |
| | (long-term) OR (long term) OR (visit to visit) OR (visit-to-visit) OR (VVV) OR (vvv) OR (intervisit) OR (between visit) OR (between-visit) OR (Visit-to-Visit) OR (Visit-to-visit) |
| | ((((((((((((((((((((((((((((((((((((((((((((((((((((((((((((((((((((gestational hypertension[MeSH Terms]) OR (preeclampsia[MeSH Terms])) OR (caesarean section[MeSH Terms])) OR (stillbirth[MeSH Terms])) OR (fetal death[MeSH Terms])) OR (premature birth[MeSH Terms])) OR (miscarriage[MeSH Terms])) OR (fetal mortality[MeSH Terms])) OR (cardiovascular pregnancy |

| | |
|---|---|
| | complication[MeSH Terms])) OR (hellp syndrome[MeSH Terms])) OR (coagulation, disseminated intravascular[MeSH Terms])) OR (placental abruption[MeSH Terms])) OR (neonatal intensive care[MeSH Terms])) OR (infant, small for gestational age[MeSH Terms])) OR (infant, low birth weight[MeSH Terms])) OR (infant, very low birth weight[MeSH Terms])) OR (fetal growth retardation[MeSH Terms])) OR (apgar score[MeSH Terms])) OR (gestational diabetes[MeSH Terms])) OR (gestational diabetes mellitus[MeSH Terms])) OR (eph gestosis[MeSH Terms])) OR (hypertension edema proteinuria gestosis[MeSH Terms])) OR (gestosis, hypertension edema proteinuria[MeSH Terms])) OR (pregnancy outcome[MeSH Terms])) OR (pregnancy outcomes[MeSH Terms])) OR (preterm birth[MeSH Terms])) OR (preterm births[MeSH Terms])) OR (complications, obstetric labor[MeSH Terms])) OR (complication, obstetric labor[MeSH Terms])) OR (maternal outcome)) OR (maternal complication)) OR (obstetric outcome)) OR (birth outcome)) OR (gestational complication)) OR (birth complication)) OR (fetal complication)) OR (neonatal complication)) OR (preterm deliver*)) OR (preterm birth)) OR (prematurity)) OR (intrauterine death)) OR (spontaneous abortion)) OR (c section*)) OR (neonatal mortalit*)) OR (neonatal morbidit*)) OR (perinatal death*)) OR (perinatal mortalit*)) OR (pregnancy-induced hypertension)) OR (maternal hypertension)) OR (preeclamptic)) OR (eclampsia[MeSH Terms])) OR (eclampsias[MeSH Terms])) OR (hemolysis elevated liver enzymes low platelet count)) OR (haemolysis elevated liver enzymes low platelet count)) OR (DIC)) OR (postpartum complication*)) OR (post-partum complication*)) OR (NICU)) OR (special care baby unit)) OR (SCBU)) OR (SGA)) OR (IUGR)) OR (LBW)) OR (VLBW)) OR (neonatal intraventricular haemorrhage)) OR (neonatal intraventricular hemorrhage)) OR (intraventricular haemorrhage of newborn)) OR (intraventricular hemorrhage of newborn) |
| | 1 AND 2 AND 3 |
| **Web of Science – Blood Pressure** | |
| | ((((((((TS=(blood pressure variabilit*)) OR TS=(blood pressure variation*)) OR TS=(diastolic variation*)) OR TS=(diastolic variabilit*)) OR TS=(systolic variation*)) OR TS=(systolic variabilit*)) OR TS=(blood pressure oscillation*)) OR TS=(blood pressure fluctuation*)) OR TS=(BPV)) |
| | ((((((((((ALL=(long-term)) OR ALL=(long term)) OR ALL=(visit to visit )) OR ALL=(visit-to-visit)) OR ALL=(VVV)) OR ALL=(vvv)) OR ALL=(intervisit )) OR ALL=(between visit)) OR ALL=(between-visit )) OR ALL=(Visit-to-Visit )) OR ALL=(Visit-to-visit ) |
| | (((((((((((((((((((((((((((((((((((((((((((((((TS=(pregnancy outcome*)) OR TS=(maternal outcome )) OR TS=(maternal complication )) OR TS=(obstetric outcome)) OR TS=(birth outcome )) OR TS=(labo$r complication*)) OR TS=(fetal complication*)) OR TS=(neonatal complication*)) OR TS=(preterm deliver*)) OR TS=(premature labo$r)) OR TS=(preterm labo$r)) OR TS=(preterm birth*)) OR TS=(premature birth*)) OR TS=(stillbirth )) OR TS=(fetal death*)) OR TS=(fetal mortalit*)) OR TS=(fetal morbidit*)) OR TS=(neonatal morbidit*)) OR TS=(neonatal mortalit*)) OR TS=(abortion*)) OR TS=(miscarriage )) OR TS=(c$esarean section*)) OR TS=(c section )) OR TS=(intrauterine death*)) OR TS=(perinatal death*)) OR TS=(perinatal mortalit*)) OR TS=(pregnancy induced hypertension )) OR TS=(maternal hypertension )) OR TS=(gestational hypertension )) OR TS=(pre-eclampsia )) OR TS=(preeclampsia )) OR TS=(pre Eclampsia )) OR TS=(preeclamptic )) OR TS=(pre Eclamptic )) OR TS=(preeclamptic tox$emia )) OR TS=(pre eclamptic tox$emia )) OR TS=(pre Eclamptic tox$emia )) OR TS=(PET )) OR TS=(pregnancy tox$emia )) OR TS=(eclampsia )) OR TS=(HELLP)) OR TS=(HELLP syndrome )) OR TS=(h$emolysis elevated liver enzymes low platelet count))) OR TS=(disseminated intravascular coagulation )) OR TS=(DIC ))) |
| | ((((((((((((((((((((((((((((TS=(placental abruption)) OR TS=(abruptio placentae)) OR TS=(postpartum complication*)) OR TS=(post-partum complication*)) OR TS=(neonatal intensive care unit )) OR TS=(NICU )) OR TS=(special care baby unit )) OR TS=(SCBU)) OR TS=(small for gestational age )) OR TS=(SGA)) OR TS=(low birth weight )) OR TS=(LBW )) OR TS=(very low birth weight )) OR |

| | |
|---|---|
| | TS=(VLBW )) OR TS=(intrauterine growth restriction )) OR TS=(intra-uterine growth restriction )) OR TS=(IUGR)) OR TS=(fetal growth restriction )) OR TS=(FGR)) OR TS=(APGAR score )) OR TS=(Apgar score )) OR TS=(neonatal intraventricular h$emorrhage )) OR TS=(gestational diabetes )) OR TS=(intraventricular h$emorrhage of newborn)) OR TS=(EPH gestosis )) OR TS=(hypertension edema proteinuria gestosis )) OR TS=(hypertension-edema-proteinuria-gestosis ))) |
| | #4 OR #3 |
| | #1 AND #2 AND #5 and English (Languages) |

## Medline and Embase (via OVID) – Heart Rate

| | |
|---|---|
| | (long-term or long term or visit to visit or visit-to-visit or VVV or vvv or intervisit or between visit or between-visit or Visit-to-Visit or Visit-to-visit).mp. [mp=ti, ab, hw, tn, ot, dm, mf, dv, kf, fx, dq, nm, ox, px, rx, an, ui, sy] |
| | exp Pregnancy Outcome/ or maternal outcome*.mp. or maternal complication*.mp. or obstetric outcome*.mp. or birth outcome*.mp. or exp Pregnancy Complications/ or gestational complication*.mp. or birth complication*.mp. or exp Obstetric Labor Complications/ or fetal complication*.mp. or neonatal complication*.mp. or preterm deliver*.mp. or preterm birth*.mp. or exp Premature Birth/ or exp Premature Birth/ or exp Obstetric Labor, Premature/ or exp Stillbirth/ or exp Fetal Death/ or exp Infant, Premature/ or prematurity.mp. or intrauterine death*.mp. or exp Abortion, Spontaneous/ or miscarriage*.mp. or exp Cesarean Section/ or c section*.mp. or exp Fetal Mortality/ or neonatal mortalit*.mp. or neonatal morbidit*.mp. or fetal morbidit*.mp. or exp Infant, Premature, Diseases/ or exp Perinatal Death/ or exp Perinatal Mortality/ or exp Hypertension, Pregnancy-Induced/ or maternal hypertension.mp. or exp Pregnancy Complications, Cardiovascular/ or gestational hypertension.mp. or exp Pre-Eclampsia/ or preeclamptic.mp. or exp Eclampsia/ or eclamptic*.mp. or exp HELLP Syndrome/ or hemolysis elevated liver enzymes low platelet count.mp. or haemolysis elevated liver enzymes low platelet count.mp. or exp Disseminated Intravascular Coagulation/ or exp Abruptio Placentae/ or placental abruption.mp. or exp Postpartum Period/ or postpartum complication*.mp. or exp Intensive Care Units, Neonatal/ or NICU.mp. or exp Intensive Care, Neonatal/ or SCBU.mp. or special care baby unit*.mp. or exp Infant, Small for Gestational Age/ or SGA.mp. or Infant, Low Birth Weight/ or LBW.mp. or intrauterine growth restriction.mp. or exp Fetal Growth Retardation/ or IUGR.mp. or exp Infant, Very Low Birth Weight/ or VLBW.mp. or exp Apgar Score/ or exp Diabetes, Gestational/ or neonatal intraventricular haemorrhage*.mp. or neonatal intraventricular hemorrhage*.mp. or intraventricular haemorrhage of newborn*.mp. or intraventricular hemorrhage of newborn*.mp. or EPH gestosis.mp. or hypertension edema proteinuria gestosis.mp. or hypertension-edema-proteinuria-gestosis.mp. |
| | exp heart rate/ or exp pulse rate/ or exp pulse/ or HRV.mp. |
| | (variabilit* or fluctuation* or oscillation* or variation).mp. |
| | 1 and 2 and 3 and 4 |
| | limit 5 to English language |
| | limit 6 to humans |

## PubMed – Heart Rate

Filters: Humans, English

| | |
|---|---|
| | ((((heart rate[MeSH Terms]) OR (pulse rate[MeSH Terms])) OR (pulse rates[MeSH Terms])) OR (pulse)) OR (HRV) OR (hrv) |
| | (((variabilit*) OR (fluctuation*)) OR (oscillation*)) OR (variation*) |
| | (long-term) OR (long term) OR (visit to visit) OR (visit-to-visit) OR (VVV) OR (vvv) OR (intervisit) OR (between visit) OR (between-visit) OR (Visit-to-Visit) OR (Visit-to-visit) |
| | (((((((((((((((((((((((((((((((((((((((((((((((((((((((((((((((((((gestational hypertension[MeSH Terms]) OR (preeclampsia[MeSH Terms])) OR (caesarean section[MeSH Terms])) OR (stillbirth[MeSH Terms])) OR (fetal death[MeSH Terms])) OR (premature birth[MeSH Terms])) OR (miscarriage[MeSH Terms])) OR (fetal mortality[MeSH Terms])) OR (cardiovascular pregnancy complication[MeSH Terms])) OR (hellp syndrome[MeSH Terms])) OR (coagulation, disseminated intravascular[MeSH Terms])) OR (placental abruption[MeSH Terms])) OR (neonatal intensive care[MeSH Terms])) OR (infant, small for gestational age[MeSH Terms])) OR (infant, low birth weight[MeSH Terms])) OR (infant, very low birth weight[MeSH Terms])) OR (fetal growth retardation[MeSH Terms])) OR (apgar score[MeSH Terms])) OR (gestational diabetes[MeSH Terms])) OR (gestational diabetes mellitus[MeSH Terms])) OR (eph gestosis[MeSH Terms])) OR (hypertension edema proteinuria gestosis[MeSH Terms])) OR (gestosis, hypertension edema proteinuria[MeSH Terms])) OR (pregnancy outcome[MeSH Terms])) OR (pregnancy outcomes[MeSH Terms])) OR (preterm birth[MeSH Terms])) OR (preterm births[MeSH Terms])) OR (complications, obstetric labor[MeSH Terms])) OR (complication, obstetric labor[MeSH Terms])) OR (maternal outcome)) OR (maternal complication)) OR (obstetric outcome)) OR (birth outcome)) OR (gestational complication)) OR (birth complication)) OR (fetal complication)) OR (neonatal complication)) OR (preterm deliver*)) OR (preterm birth)) OR (prematurity)) OR (intrauterine death)) OR (spontaneous abortion)) OR (c section*)) OR (neonatal mortalit*)) OR (neonatal morbidit*)) OR (perinatal death*)) OR (perinatal mortalit*)) OR (pregnancy-induced hypertension)) OR (maternal hypertension)) OR (preeclamptic)) OR (eclampsia[MeSH Terms])) OR (eclampsias[MeSH Terms])) OR (hemolysis elevated liver enzymes low platelet count)) OR (haemolysis elevated liver enzymes low platelet count)) OR (DIC)) OR (postpartum complication*)) OR (post-partum complication*)) OR (NICU)) OR (special care baby unit)) OR (SCBU)) OR (SGA)) OR (IUGR)) OR (LBW)) OR (VLBW)) OR (neonatal intraventricular haemorrhage)) OR (neonatal intraventricular hemorrhage)) OR (intraventricular haemorrhage of newborn)) OR (intraventricular hemorrhage of newborn) |
| | 1 AND 2 AND 3 AND 4 |

## Web of Science – Heart Rate

| | |
|---|---|
| | ((((TS=(heart rate)) OR TS=(pulse rate)) OR TS=(pulse)) OR TS=(HRV)) OR TS=(hrv) |
| | (((((((((((ALL=(long-term)) OR ALL=(long term)) OR ALL=(visit to visit )) OR ALL=(visit-to-visit)) OR ALL=(VVV)) OR ALL=(vvv)) OR ALL=(intervisit )) OR ALL=(between visit)) OR ALL=(between-visit )) OR ALL=(Visit-to-Visit )) OR ALL=(Visit-to-visit )) |

| | |
|---|---|
| | ((((((((((((((((((((((((((((((((((((((((((((((((TS=(pregnancy outcome*)) OR TS=(maternal outcome )) OR TS=(maternal complication )) OR TS=(obstetric outcome)) OR TS=(birth outcome )) OR TS=(labo$r complication*)) OR TS=(fetal complication*)) OR TS=(neonatal complication*)) OR TS=(preterm deliver*)) OR TS=(premature labo$r)) OR TS=(preterm labo$r)) OR TS=(preterm birth*)) OR TS=(premature birth*)) OR TS=(stillbirth )) OR TS=(fetal death*)) OR TS=(fetal mortalit*)) OR TS=(fetal morbidit*)) OR TS=(neonatal morbidit*)) OR TS=(neonatal mortalit*)) OR TS=(abortion*)) OR TS=(miscarriage )) OR TS=(c$esarean section*)) OR TS=(c section )) OR TS=(intrauterine death*)) OR TS=(perinatal death*)) OR TS=(perinatal mortalit*)) OR TS=(pregnancy induced hypertension )) OR TS=(maternal hypertension )) OR TS=(gestational hypertension )) OR TS=(pre-eclampsia )) OR TS=(preeclampsia )) OR TS=(pre Eclampsia )) OR TS=(preeclamptic )) OR TS=(pre Eclamptic )) OR TS=(preeclamptic tox$emia )) OR TS=(pre eclamptic tox$emia )) OR TS=(pre Eclamptic tox$emia )) OR TS=(PET )) OR TS=(pregnancy tox$emia )) OR TS=(eclampsia )) OR TS=(HELLP)) OR TS=(HELLP syndrome )) OR TS=(h$emolysis elevated liver enzymes low platelet count))) OR TS=(disseminated intravascular coagulation )) OR TS=(DIC )))) |
| | (((((((((((((((((((((((((((((TS=(placental abruption)) OR TS=(abruptio placentae)) OR TS=(postpartum complication*)) OR TS=(post-partum complication*)) OR TS=(neonatal intensive care unit )) OR TS=(NICU )) OR TS=(special care baby unit )) OR TS=(SCBU)) OR TS=(small for gestational age )) OR TS=(SGA)) OR TS=(low birth weight )) OR TS=(LBW )) OR TS=(very low birth weight )) OR TS=(VLBW )) OR TS=(intrauterine growth restriction )) OR TS=(intra-uterine growth restriction )) OR TS=(IUGR)) OR TS=(fetal growth restriction )) OR TS=(FGR)) OR TS=(APGAR score )) OR TS=(Apgar score )) OR TS=(neonatal intraventricular h$emorrhage )) OR TS=(gestational diabetes )) OR TS=(intraventricular h$emorrhage of newborn)) OR TS=(EPH gestosis )) OR TS=(hypertension edema proteinuria gestosis )) OR TS=(hypertension-edema-proteinuria-gestosis )))) |
| | (((ALL=(variabilit*)) OR ALL=(fluctuation*)) OR ALL=(oscillation)) OR ALL=(variation*) |
| | 3 OR 4 |
| | 1 AND 2 AND 5 AND 6 and English (Languages) |

| **Table S3**. Characteristics of Included Studies | | | | | | | | | |
|---|---|---|---|---|---|---|---|---|---|
| Study | Data Collection Period | Country | Participants (N) | Design | Subgroups | Inclusions | Exclusions | Adjustment factors | Outcomes |
| Kim et al., 2018[7] | 2001 - 2010 | South Korea | 4,163 | Retrospective observational | Normotensive (n=3,679), gestational hypertension (n=74), pre-eclampsia (n=410) | Patients with gestational hypertension, and with sufficient BP data to calculate VVV at three time points | Chronic hypertension, proteinuria before 20 weeks | Mean sBP for relevant gestational age (at BP measurement) Maternal age, BMI, weight gain, presence of multiple pregnancies | Pregnancy composite**†** |
| Jieyu et al., 2019[8] | 2015 - 2018 | China | 14,702 | Retrospective observational | Normotensive (n=13,693), pre-eclampsia (n=131), gestational hypertension (n=878) | ≥3 BP measurements in the second or third trimester | Hypertension or proteinuria at initial antenatal examination, polyembryony | Mean sBP, mean dBP, Maternal age, BMI, weight gain, BP measurement times at each trimester or during the whole pregnancy, gestational age, presence of GDM, anemia, gestation and parturition | Gestational hypertension and pre-eclampsia |
| Magee et al., 2020[9] | 2010 - 2014 | 94 centres in 15 countries***** | 913 | Retrospective observational | Less tight (n=464) vs tight control (n=449) of hypertension groups from CHIPS | Non-proteinuric chronic or gestational hypertension | <2 BP measurements | Mean post-randomisation BP Treatment group (tight vs. less tight), gestational age at randomisation, centre, hypertension type, antihypertensive at randomisation, prior severe hypertension in this | Maternal composite**‡**, severe hypertension, pre-eclampsia (broad definition), pre-eclampsia (restrictive definition), composite perinatal outcome**§**, |

| | | | | | | | | | |
|---|---|---|---|---|---|---|---|---|---|
| | | | | | | | | pregnancy, gestational diabetes | birthweight <10th centile, delivery at <34 weeks, delivery at <37 weeks |
| Liu et al., 2020[10] | 2014 - 2019 | China | 48,209 | Retrospective observational | Maternal age (years) (<24 [8,681], 24-31 [25,681], >=31 [13,847], early pregnancy BMI (kg/m2) (<18 [4,472], 18-24 [34,323], >=24 [9,414]), HDP (894), GDM (6,878), primipara (14,166), full-term delivery (28,602), infant sex (male [24,660]), study city (Taizhou [32,447], Taicang [15,762]) | Singleton pregnancy, ≥3 BP measurements from 20 weeks' gestation | Chronic hypertension or proteinuria at booking | Mean sBP, mean dBP (or MAP)<br>Maternal age, BMI, parity<br>infant sex, , gestation, , GDM status, anemia, thyroid disease, GH and PE/eclampsia, abortion during previous pregnancy, number of BP measurements after 20 weeks' gestation, and study city | SGA, LBW |
| Magee et al., 2021[11] | 2014-2017 | India, Pakistan, and Mozambique | 17, 770 | Retrospective observational | India (n=5,355), Pakistan (n=8,895), Mozambique (n=3,520) | CLIP women with ≥2 antenatal care contacts | Participants with <2 antenatal care contacts | Mean BP<br>Maternal age, parity, basic education, gestational age at enrolment, country and cluster | Hypertension, primary composite**#**, maternal composite******, maternal mortality, maternal morbidity, perinatal composite**††**, perinatal stillbirth, early NND, late NND, |

| | | | | | | | | | |
|---|---|---|---|---|---|---|---|---|---|
| | | | | | | | | | neonatal morbidity |
| Tadic et al., 2021(a)[12] | 2021 | Serbia | 161 | Cross-sectional | Normotensive (n=56), gestational hypertension (n=55), pre-eclampsia (n=50) | Normotensive controls of similar age and GA to included patients with gestational hypertension and pre-eclampsia | Patients with congenital heart disease, valvular heart disease, coronary artery disease, pulmonary disease, pregestational diabetes | NA | Gestational hypertension and pre-eclampsia |
| Tadic et al., 2021(b)[13] | 2021 | Serbia | 140 | Cross-sectional | Normotensive (n=45), pre-eclampsia (n=45), gestational hypertension (n=50) | Normotensive controls of similar age and GA to included patients with gestational hypertension and pre-eclampsia | Patients with congenital heart disease, valvular heart disease, coronary artery disease, pulmonary disease, pregestational diabetes | NA | Gestational hypertension and pre-eclampsia |
| Gu et al., 2022[14] | 2017 - 2020 | China | 52,891 | Retrospective observational | Fetal distress (10,445), SGA (1422), PTB (2402), 1-min Apgar score <7 (685), 5-min Apgar score <7 (67) | ≥6 BP measurements during pregnancy | Stillbirth, pre-gestational diabetes, placenta previa, proteinuria <20 weeks', renal disease, chronic hypertension | Mean sBP, mean dBP, Maternal age, BMI, parity, gravidity, cesarean, IVF transfer, HDP, GDM, premature rupture of membranes, cephalic presentation, | Fetal distress, SGA, PTB, 1-min Apgar score <7, 5-min Apgar score <7 |

| | | | | | | | | | |
|---|---|---|---|---|---|---|---|---|---|
| | | | | | | | | scarred uterus, anemia | |

ARV indicates average real variability; BP, blood pressure; BMI, body mass index; CHIPS, Control of Hypertension in Pregnancy Study; CLIP, Community-Level Interventions for Pre-eclampsia; CV, coefficient of variation; GA, gestational age; GDM, gestational diabetes mellitus; HDP, hypertensive disorders of pregnancy; LBW, low birth weight; NND, neonatal death; PTB, preterm birth; SD, standard deviation; SGA, small-for-gestational-age.

***** Argentina, Australia, Brazil, Canada, Chile, Columbia, Estonia, Hungary, Israel, Jordan, New Zealand, Poland, The Netherlands, United Kingdom, USA.

**†** Delivery method, placental abruptio, preterm labour, pulmonary oedema, deep vein thrombosis, pulmonary thromboembolism, HELLP syndrome, heart failure, maternal death, 1-min and 5-min Apgar scores, birth weight, meconium, respiratory distress, fetal death.

**‡** Serious maternal complications before 6 weeks postpartum or until hospital discharge, whichever was later; serious maternal complications included death, stroke, eclampsia, blindness, uncontrolled hypertension, the use of inotropic agents, pulmonary oedema, respiratory failure, myocardial ischemia or infarction, hepatic dysfunction, hepatic haematoma or rupture, renal failure, and transfusion.

**§** Pregnancy-loss or high-level neonatal care (greater than normal newborn care) for ≥48hours in the first 28 days of life.

**#** Composite of maternal or perinatal mortality or morbidity (primary outcome).

****** Mortality or morbidity.

**††** Stillbirth, early or late neonatal death, or neonatal morbidity.

| **Table S4**. Blood Pressure Measurement Characteristics | | | | | | |
|---|---|---|---|---|---|---|
| Study | Metrics | sBP/dBP | BP Measurement Method | No. Of BP Measurements | BPV Calculation | Continuous vs Categorical |
| Kim et al., 2018[7] | SD + CV | sBP + dBP | Automated oscillometric device, seated position, after 5 minutes rest | 3 measurements for each timepoint (at 10-, 20- and 30-weeks' gestation) | ≥3 BP measurements obtained on different visits at each of 10-, 20- and 30- week timepoint if the interval between visits was less than 2 weeks | Continuous |
| Jieyu et al., 2019[8] | SD + CV | sBP + dBP | Mercury sphygmomanometer, seated position, after 5 minutes rest | 3.91 ± 0.44 (2nd trimester), 5.46 ± 1.51 (3rd trimester), 9.37 ± 1.60 (whole pregnancy) | ≥3 BP measurements in the second trimester and ≥3 BP measurements in the third trimester. | Continuous |
| Magee et al., 2020[9] | SD + ARV | sBP + dBP | Aneroid, automated and mercury sphygmomanometers, after 5 minutes rest, average of 2nd and 3rd BP readings was recorded | 74.5% of women had <=4 measurements | Using all office/clinic visits after randomisation until delivery, except for BP at randomisation (≥2) | Continuous |
| Liu et al., 2020[10] | SD + CV | MAP + sBP + dBP | Mercury sphygmomanometer, seated position, after 5 minutes rest | 7 [5–8] | Using ≥3 BP measurements after 20 weeks gestation | Categorical (four quartiles) |
| Magee et al., 2021[11] | SD + ARV | sBP + dBP | Semiautomated pregnancy- and preeclampsia-validated oscillometric device (Microlife 3AS1-2), after 5 | 5 [3–7] | Using all CLIP contacts after enrolment until delivery (POM contacts | Continuous |

**Table S4**. Blood Pressure Measurement Characteristics

| Study | Metrics | sBP/dBP | BP Measurement Method | No. Of BP Measurements | BPV Calculation | Continuous vs Categorical |
|---|---|---|---|---|---|---|
| | | | minutes rest, average of the 2nd and 3rd readings recorded | | every 4 weeks minimum) (≥2) | |
| Tadic et al., 2021(a)[12] | SD + CV | sBP + dBP | Calibrated sphygmomanometer, seated position, average of two consecutive measurements was recorded | *NA* | Using all clinic visits from baseline until delivery | Continuous |
| Tadic et al., 2021(b)[13] | SD + ARV + CV | sBP | Calibrated sphygmomanometer, seated position, average of two consecutive measurements was recorded | *NA* | Using all systolic BPs from baseline until delivery | Continuous |
| Gu et al., 2022[14] | SD + CV | sBP + dBP | Calibrated sphygmomanometer, seated position, after 5 minutes rest | 11.51 ± 2.14 | Using all blood pressure measurements taken from antenatal visits (≥6) | Categorical (four quartiles) |

ARV, average real variability; BPV, blood pressure variability; CV, coefficient of variation; dBP, diastolic blood pressure; MAP, mean arterial pressure; sBP, systolic blood pressure; SD, standard deviation, VVV; visit-to-visit variability.

| **Table S5**. QUADAS-2 Tool - Risk of Bias Assessment | | | | | | | |
|---|---|---|---|---|---|---|---|
| Study | Risk of Bias | | | | Applicability Concerns | | |
| | Patient Selection | Index Test | Reference Standard | Flow and Timing | Patient Selection | Index Test | Reference Standard |
| Kim et al., 2018[7] | High – Excluded women with chronic hypertension and proteinuria at baseline. | Low | Low | Low | Low | Low | Low |
| Jieyu et al., 2019[8] | High – Excluded women with chronic hypertension and proteinuria at baseline. | Low | Low | Low | Low | Low | Low |
| Magee et al., 2020[9] | Low | Low | Low | Low | Low | Low | Low |
| Liu et al., 2020[10] | High – Excluded women with chronic hypertension and proteinuria at baseline. | Low | Low | Low | Low | Low | Low |
| Magee et al., 2021[11] | Low | Low | Low | Low | Low | Low | Low |
| Tadic et al., 2021(a)[12] | Low | Low | Low | Low | Low | Low | Low |
| Tadic et al., 2021(b)[13] | Low | Low | Low | Low | Low | Low | Low |
| Gu et al., 2022[14] | High – Excluded women with chronic hypertension and proteinuria at baseline. | Low | Low | Low | Low | Low | Low |

**Table S6**. Participant Characteristics

Either mean ± SD, median [IQR] or N (%)

| Study | Maternal Age (years) | Maternal BMI ($kg/m^2$) | Ethnicity | Primiparous | Multiple pregnancy | Antihypertensive therapy among hypertensive women | GA at delivery (weeks) |
|---|---|---|---|---|---|---|---|
| Kim et al., 2018[7] | 32.6 ± 3.5 | 21.2 ± 3.0 | *NA* | 2624 (63.0) | 148 (3.6) | *NA* | 39.1 ± 1.4 |
| Jieyu et al., 2019[8] | 27.15 ± 4.32 | 21.54 ± 3.14 | *NA* | 7672 (52.18) | 0 | *NA* | 37.34 ± 1.64 |
| Magee et al., 2020[9] | 34.4 [30.0-38.2] | 30.0 [25.4-35.7] | Caucasian/Asian/Other: 688 (75.4) Black/Hispanic: 225 (24.6) | 300 (32.9) | 0 | 519 (56.8)***** | *NA* |
| Liu et al., 2020[10] | 27.98 ± 4.86 | 21.58 ± 3.18 | *NA* | 14166 (29.38) | 0 | *NA* | 38.70 ± 1.31 |
| Magee et al., 2021[11] | 25 [22-30] | *NA* | *NA* | 5032 (28.3) | 0 | *NA* | 39.0 [37.0-40.4] |
| Tadic et al., 2021(a)[12] | 30.65 ± 4.82 | 29.37 ± 7.18 | *NA* | (Median [IQR parity: 1 [1–3]) | 0 | 46 (43.8) | *NA* |
| Tadic et al., 2021(b)[13] | 31 ± 6.24 | 29.82 ± 7.48 | *NA* | (Median [IQR parity: 1 [1–3]) | 0 | 40 (42.0) | *NA* |
| Gu et al., 2022[14] | 31.27 ± 4.00 | 21.22 ± 2.67 | *NA* | (Mean±SD parity: 1.32 ± 0.49) | 0 | *NA* | *NA* |

*****At randomisation

**Table S7**. Blood Pressure Variability
(Either mean ± SD, or median [IQR])

| Study | Timepoints | Systolic SD | Diastolic SD | Systolic ARV | Diastolic ARV | Systolic CV | Diastolic CV |
|---|---|---|---|---|---|---|---|
| **Normotensive Participants** | | | | | | | |
| Study | Timepoints | Systolic SD | Diastolic SD | Systolic ARV | Diastolic ARV | Systolic CV | Diastolic CV |
| Kim et al., 2018[7] | 10 weeks* | 7.2 ± 4.2 | *NA* | *NA* | *NA* | 6.4 ± 3.8 | *NA* |
| | 20 weeks* | 6.8 ± 3.9 | | | | 6.0 ± 3.5 | |
| | 30 weeks* | 6.3 ± 3.6 | | | | 5.5 ± 3.2 | |
| | Overall | 6.7 ± 4.0 | | | | 6.0 ± 3.6 | |
| Jieyu et al., 2019[8] | Baseline to delivery | 8.38 ± 2.70 | 6.42 ± 2.05 | *NA* | *NA* | 7.46 ± 2.46 | 9.36 ± 3.11 |
| | 2nd trimester | 6.79 ± 3.30 | *NA* | | | 6.22 ± 3.03 | *NA* |
| | 3rd trimester | 7.29 ± 3.17 | *NA* | | | 6.35 ± 2.82 | *NA* |
| Tadic et al., 2021(a)[12] | Baseline to delivery | 8.0 ± 1.9 | *NA* | *NA* | *NA* | 7.6 ± 1.3 | *NA* |
| Tadic et al., 2021(b)[13] | Baseline to delivery | 8.5 ± 1.5 | *NA* | 6.8 ± 3.1 | *NA* | 7.3 ± 1.3 | *NA* |
| Gu et al., 2022[14] | Baseline to delivery | 7.80 ± 2.65 | 6.25 ± 2.45 | *NA* | *NA* | 7.04 ± 2.43 | 9.11 ± 3.67 |
| **Median [IQR]** | Overall | 8.00 [7.80-8.38] | 6.34 [6.29-6.38] | *NA* | *NA* | 7.30 [7.04-7.46] | 9.26 [9.17-9.30] |
| **Whole Cohort** | | | | | | | |
| Study | Timepoints | Systolic SD | Diastolic SD | Systolic ARV | Diastolic ARV | Systolic CV | Diastolic CV |
| Liu et al., 2020[10] | After 20 weeks' gestation | 7.29 [5.50–9.37] | 5.46 [4.10–7.09] | *NA* | *NA* | 6.42 [4.83–8.26] | 8.02 [6.02–10.36] |
| | *Mean ± SD†* | 7.39 ± 2.87 | 5.55 ± 2.22 | *NA* | *NA* | 6.50 ± 2.54 | 8.13 ± 3.22 |
| Magee et al., 2021[11] | Baseline to delivery | 6.27 [4.19, 8.77] | 5.28 [3.54, 7.50] | 6.67 [4.43, 9.83] | 5.67 [3.75, 8.20] | *NA* | *NA* |
| | *Mean ± SD†* | 6.41 ± 3.40 | 5.44 ± 2.94 | 6.98 ± 4.00 | 5.87 ± 3.30 | *NA* | *NA* |
| Gu et al., 2022[14] | Baseline to delivery | 7.88 ± 2.69 | 6.30 ± 2.47 | *NA* | *NA* | 7.06 ± 2.43 | 9.10 ± 3.66 |
| **Median [IQR]** | Overall | 7.39 [6.90-7.64] | 5.55 [5.50-5.93] | *NA* | *NA* | 6.78 [6.64-6.92] | 8.62 [8.37-8.86] |
| **Hypertensive Participants** | | | | | | | |
| Study | Timepoints | Systolic SD | Diastolic SD | Systolic ARV | Diastolic ARV | Systolic CV | Diastolic CV |
| | 10 weeks* | 8.2 ± 5.7 | *NA* | *NA* | *NA* | 6.7 ± 4.4 | *NA* |

| | | | | | | | |
|---|---|---|---|---|---|---|---|
| Kim et al., 2018[7] | 20 weeks* | 7.6 ± 5.0 | *NA* | *NA* | *NA* | 6.3 ± 4.2 | *NA* |
| | 30 weeks* | 8.3 ± 5.3 | *NA* | *NA* | *NA* | 6.5 ± 4.0 | *NA* |
| | Overall | 8.0 ± 5.4 | *NA* | *NA* | *NA* | 6.5 ± 4.2 | |
| Jieyu et al., 2019[8] | Baseline to delivery | 10.73 ± 3.33 | 7.93 ± 2.52 | *NA* | *NA* | 8.76 ± 2.94 | 10.46 ± 3.62 |
| | 2nd trimester | 7.62 ± 3.90 | *NA* | *NA* | *NA* | 6.51 ± 3.34 | *NA* |
| | 3rd trimester | 9.30 ± 4.13 | *NA* | *NA* | *NA* | 7.35 ± 3.45 | *NA* |
| Magee et al., 2020[9] | Baseline to delivery | 9.0 [6.7-11.6] | 6.6 [4.7-8.7] | 9.2 [6.8-12.1] | 6.9 [4.6-9.3] | *NA* | *NA* |
| | *Mean ± SD†* | 9.1 ± 3.6 | 6.7 ± 3.0 | 9.37 ± 3.94 | 6.93 ± 3.49 | *NA* | *NA* |
| Magee et al., 2021[11] | Baseline to delivery | 11.56 [7.39 - 16.00] | 10.56 [7.68-13.99] | 10.11 [7.00-14.67] | 9.30 [6.49-14.00] | *NA* | *NA* |
| | *Mean ± SD†* | 11.65 ± 6.38 | 10.74 ± 4.68 | 10.59 ± 5.69 | 9.93 ± 5.57 | *NA* | *NA* |
| Tadic et al., 2021(a)[12] | Baseline to delivery | 15.3 ± 3.3 | *NA* | *NA* | *NA* | 13.1 ± 2.3 | *NA* |
| Tadic et al., 2021(b)[13] | Baseline to delivery | 14.0 ± 3.5 | *NA* | 10.5 ± 4.2 | *NA* | 10.2 ± 2.1 | *NA* |
| Gu et al., 2022[14]‡ | Baseline to delivery | 9.12 ± 3.02 | 7.13 ± 2.58 | *NA* | *NA* | 7.33 ± 2.47 | 9.02 ± 3.54 |
| **Median [IQR]** | Overall | 10.73 [9.11-12.82] | 7.53 [7.02-8.63] | 10.50 [9.94-10.55] | 8.43 [7.68-9.18] | 8.76 [7.33-10.20] | 9.74 [9.38-10.10] |
| **Participants with preeclampsia** | | | | | | | |
| Study | Timepoints | Systolic SD | Diastolic SD | Systolic ARV | Diastolic ARV | Systolic CV | Diastolic CV |
| Jieyu et al., 2019[8] | Baseline to delivery | 9.56 ± 3.69 | 6.72 ± 2.34 | *NA* | *NA* | 8.21 ± 3.06 | 9.39 ± 3.27 |
| | 2nd trimester | 7.62 ± 3.96 | *NA* | *NA* | *NA* | 6.82 ± 3.51 | *NA* |
| | 3rd trimester | 8.03 ± 4.02 | *NA* | *NA* | *NA* | 6.77 ± 3.35 | *NA* |
| Tadic et al., 2021(a)[12] | Baseline to delivery | 12.8 ± 3.1 | *NA* | *NA* | *NA* | 11.0 ± 2.0 | *NA* |
| Tadic et al., 2021(b)[13] | Baseline to delivery | 12.4 ± 3.3 | *NA* | 9.4 ± 3.7 | *NA* | 9.3 ± 1.9 | *NA* |
| Gu et al., 2022[14]‡ | Baseline to delivery | 9.12 ± 3.02 | 7.13 ± 2.58 | *NA* | *NA* | 7.33 ± 2.47 | 9.02 ± 3.54 |

| | | | | | | | |
|---|---|---|---|---|---|---|---|
| **Median [IQR]** | Overall | 10.98 [9.45-12.5] | 6.93 [6.82-7.03] | *NA* | *NA* | 8.76 [7.99-9.73] | 9.21 [9.11-9.30] |

ARV indicates average real variability; CV, coefficient of variation; SD, standard deviation.

***** BPV was evaluated using three BP measurements from different visits around each time point, if the interval between visits was less than two weeks.

**†** Mean and variance estimated from the median, range, and sample size.

**‡** Numbers are for women with gestational hypertension and pre-eclampsia combined.

**Table S8**. Maternal Outcomes
(odds ratio and 95% confidence interval unless otherwise stated)

| | | | | | | | | |
|---|---|---|---|---|---|---|---|---|
| **All Participants** | | | | | | | | |
| | Study | Gestational hypertension | Preeclampsia | Severe hypertension | Pregnancy composite | Maternal composite | Maternal morbidity | Maternal mortality |
| **sBP SD** | **Kim et al., 2018[7]** | | | | | | | |
| | 10 weeks | | | | 1.21 [1.02-1.44] | | | |
| | 20 weeks | | | | 1.04 [0.87-1.23] | | | |
| | 30 weeks | | | | 1.25 [1.05-1.49] | | | |
| | Pooled | | | | 1.16 [1.04-1.30] | | | |
| | **Jieyu et al., 2019[8]** | | | | | | | |
| | 2nd trimester | 1.05 [1.03-1.08] | 1.06 [1.01-1.11] | | | | | |
| | 3rd trimester | 1.34 [1.31-1.38] | 1.06 [1.01-1.11] | | | | | |
| | Overall | 1.49 [1.44-1.54] | 1.13 [1.06-1.19] | | | | | |
| | **Magee et al., 2021[11]** | | | | | | | |
| | Overall | 1.78 [1.70-1.88] | | 2.20 [1.98-2.46] | 1.10 [1.06-1.14] | 1.08 [1.03-1.14] | 1.08 [1.02-1.13] | 1.23 [0.96-1.59] |
| **sBP ARV** | **Magee et al., 2021[11]** | | | | | | | |
| | Overall | 1.40 [1.34-1.47] | | 1.51 [1.36-1.67] | 1.06 [1.02-1.10] | 1.04 [0.99-1.10] | 1.04 [0.99-1.09] | 1.19 [0.94-1.50] |
| **sBP CV** | **Kim et al., 2018[7]** | | | | | | | |
| | 10 weeks | | | | 1.21 [1.02-1.44] | | | |
| | 20 weeks | | | | 1.02 [0.86-1.21] | | | |
| | 30 weeks | | | | 1.17 [0.98-1.39] | | | |
| | Pooled | | | | 1.13 [1.02-1.25] | | | |
| | **Jieyu et al., 2019[8]** | | | | | | | |
| | 2nd trimester | 1.08 [1.05-1.10] | 1.07 [1.01-1.12] | | | | | |
| | 3rd trimester | 1.41 [1.37-1.45] | 1.07 [1.01-1.13] | | | | | |
| | Overall | 1.62 [1.56-1.68] | 1.14 [1.06-1.21] | | | | | |
| **dBP SD** | **Jieyu et al., 2019[8]** | | | | | | | |
| | 2nd trimester | 1.05 [1.03-1.08] | 1.04 [0.97-1.10] | | | | | |
| | 3rd trimester | 1.36 [1.32-1.40] | 0.99 [0.93-1.07] | | | | | |
| | Overall | 1.57 [1.51-1.63] | 1.05 [0.97-1.14] | | | | | |
| | **Magee et al., 2021[11]** | | | | | | | |

| | | | | | | | | |
|---|---|---|---|---|---|---|---|---|
| | Overall | 2.15 [2.01-2.27] | | 1.98 [1.79-2.20] | 1.07 [1.03-1.11] | 1.08 [1.02-1.13] | 1.08 [1.02-1.13] | 1.39 [1.14-1.70] |
| **dBP ARV** | **Magee et al., 2021[11]** | | | | | | | |
| | Overall | 1.65 [1.57-1.73] | | 1.40 [1.26, 1.55] | 1.06 [1.02-1.09] | 1.05 [1.00-1.11] | 1.05 [1.00-1.10] | 1.35 [1.14-1.60] |
| **dBP CV** | **Jieyu et al., 2019[8]** | | | | | | | |
| | 2nd trimester | 1.05 [1.03-1.07] | 1.02 [0.98-1.07] | | | | | |
| | 3rd trimester | 1.24 [1.21-1.27] | 1.00 [0.95-1.05] | | | | | |
| | Overall | 1.40 [1.36-1.44] | 1.03 [0.97-1.09] | | | | | |
| **Hypertensive Participants** | | | | | | | | |
| | Study | Gestational hypertension | Preeclampsia | Severe hypertension | Pregnancy composite | Maternal composite | Maternal morbidity | Maternal mortality |
| **sBP SD** | **Magee et al., 2020[9]** | | | | | | | |
| | Overall | | 1.23 [1.04-1.44] | 1.80 [1.47-2.20] | | 1.27 [0.91-1.78] | | |
| | **Magee et al., 2021[11]** | | | | | | | |
| | | | | | 1.32 [1.12-1.56] | 1.26 [1.02-1.56] | 1.23 [0.98-1.53] | 2.07 [1.14-3.76] |
| | Study | Gestational hypertension | Preeclampsia | Severe hypertension | Pregnancy composite | Maternal composite | Maternal morbidity | Maternal mortality |
| **sBP ARV** | **Magee et al., 2020[9]** | | | | | | | |
| | Overall | | 1.29 [1.10-1.53] | 1.60 [1.32-1.94] | | 1.25 [0.92-1.69] | | |
| | **Magee et al., 2021[11]** | | | | | | | |
| | Overall | | | | 1.14 [0.96-1.35] | 1.02 [0.82-1.28] | 0.98 [0.77-1.25] | 1.69 [1.05-2.71] |
| **sBP CV** | No studies reported maternal outcomes in hypertensive women with sBP CV | | | | | | | |
| | Study | Gestational hypertension | Preeclampsia | Severe hypertension | Pregnancy composite | Maternal composite | Maternal morbidity | Maternal mortality |
| **dBP SD** | **Magee et al., 2020[9]** | | | | | | | |
| | Overall | | 1.33 [1.13-1.56] | 1.42 [1.17-1.71] | | 1.25 [0.88-1.75] | | |
| | **Magee et al., 2021[11]** | | | | | | | |
| | Overall | | | | 1.21 [1.03-1.43] | 1.28 [0.98-1.67] | 1.40 [1.12-1.76] | 1.99 [0.74-5.37] |
| | Study | Gestational hypertension | Preeclampsia | Severe hypertension | Pregnancy composite | Maternal composite | Maternal morbidity | Maternal mortality |

| | | | | | | | | |
|---|---|---|---|---|---|---|---|---|
| dBP ARV | **Magee et al., 2020[9]** | | | | | | | |
| | Overall | | 1.32 [1.11-1.57] | 1.29 [1.07-1.57] | | 1.17 [0.86-1.61] | | |
| | **Magee et al., 2021[11]** | | | | | | | |
| | Overall | | | | 1.09 [0.92-1.29] | 1.20 [0.97-1.48] | 1.21 [0.97-1.50] | 1.97 [0.94-4.12] |
| dBP CV | No studies reported maternal outcomes in hypertensive women with dBP CV | | | | | | | |

ARV indicates average real variability; BP, blood pressure; CI, confidence interval; CV, coefficient of variation; dBP, diastolic blood pressure; OR, odds ratio; sBP, systolic blood pressure; SD, standard deviation.

ORs for association between adverse maternal outcomes and measures of BP variability in hypertensive participants.

**Table S9**. Perinatal Outcomes

| | Study | PTB <37 weeks | SGA | Perinatal composite | Stillbirth | Neonatal morbidity | Late NND | Early NND |
|---|---|---|---|---|---|---|---|---|
| **All Participants** | | | | | | | | |
| **sBP SD** | **Magee et al., 2021[11]** | | | | | | | |
| | Overall | 1.01 (0.98, 1.06) | | 1.08 (1.04-1.13) | 1.12 (1.04-1.20) | 1.09 (1.04-1.15) | 1.11 (0.95-1.30) | 0.98 (0.90-1.07) |
| **sBP ARV** | Overall | 1.07 (1.03, 1.11) | | 1.06 (1.02-1.11) | 1.12 (1.05-1.20) | 1.05 (1.00-1.10) | 1.07 (0.91-1.25) | 1.00 (0.92-1.09) |
| **sBP CV** | No studies reported perinatal outcomes with sBP CV | | | | | | | |
| **dBP SD** | Overall | 0.99 (0.95, 1.03) | | 1.05 (1.01-1.09) | 1.10 (1.03-1.18) | 1.05 (1.00-1.10) | 1.03 (0.88-1.20) | 0.98 (0.90-1.07) |
| **dBP ARV** | Overall | 1.05 (1.02, 1.10) | | 1.05 (1.01-1.10) | 1.12 (1.05-1.20) | 1.02 (0.97-1.08) | 1.03 (0.89-1.20) | 1.00 (0.92-1.09) |
| **dBP CV** | No studies reported perinatal outcomes with dBP CV | | | | | | | |
| | **Hypertensive Participants** | | | | | | | |
| | Study | PTB <37 weeks | SGA | Perinatal composite | Stillbirth | Neonatal morbidity | Late NND | Early NND |

| | | | | | | | | |
|---|---|---|---|---|---|---|---|---|
| **sBP SD** | **Magee et al., 2020[9]** | | | | | | | |
| | Overall | 1.18 (1.00-1.39) | 1.03 (0.86-1.23) | 1.05 (0.89-1.24) | 1.23 (0.79, 1.92) | 1.03 (0.87, 1.22) | | |
| | **Magee et al., 2021[11]** | | | | | | | |
| | Overall | | | 1.19 (0.93-1.52) | 1.08 (0.76-1.52) | 1.21 (0.89-1.66) | 1.24 (0.98-1.56) | 1.32 (0.71-2.42) |
| **sBP ARV** | **Magee et al., 2020[9]** | | | | | | | |
| | Overall | 1.21 (1.03-1.43) | 1.00 (0.85-1.19) | 1.17 (0.99-1.38) | 1.48 (1.03-2.13) | 1.13 (0.96-1.33) | | |
| | **Magee et al., 2021[11]** | | | | | | | |
| | Overall | | | 1.13 (0.95-1.36) | 1.05 (0.83-1.35) | 1.29 (1.02-1.63) | 1.39 (0.85-2.25) | 1.03 (0.74-1.44) |
| **sBP CV** | No studies reported perinatal outcomes in hypertensive women with sBP CV | | | | | | | |
| **dBP SD** | **Magee et al., 2020[9]** | | | | | | | |
| | Overall | 0.90 (0.76-1.07) | 0.84 (0.70-1.01) | 0.85 (0.72-1.01) | 1.08 (0.68, 1.72) | 0.84 (0.70, 1.00) | | |
| | **Magee et al., 2021[11]** | | | | | | | |
| | Overall | | | 1.09 (0.91-1.31) | 1.05 (0.80-1.36) | 1.14 (0.89-1.45) | 0.8 (0.40-1.62) | 1.13 (0.80-1.61) |
| **dBP ARV** | **Magee et al., 2020[9]** | | | | | | | |
| | Overall | 0.95 (0.81-1.12) | 0.88 (0.73-1.06) | 0.93 (0.78-1.10) | 1.29 (0.91, 1.83) | 0.89 (0.75, 1.06) | | |
| | **Magee et al., 2021[11]** | | | | | | | |
| | Overall | | | 1.00 (0.82, 1.21) | 0.95 (0.71, 1.26) | 1.13 (0.89, 1.45) | 0.99 (0.49, 1.97) | 1.03 (0.71, 1.50) |

| | |
|---|---|
| **dBP CV** | No studies reported perinatal outcomes in hypertensive women with dBP CV |

ARV indicates average real variability; BP, blood pressure; CV, coefficient of variation; dBP, diastolic blood pressure; LBW, low birth weight; NND, neonatal death; PTB, preterm birth; sBP, systolic blood pressure; SGA, small-for-gestational-age.

ORs for association between adverse perinatal outcomes and measures of BP variability in hypertensive participants.

| **Table S10**. Perinatal Outcomes in All Participants (Quartiles of BP Variability) | | | | | |
|---|---|---|---|---|---|
| **PTB** | | 1st Quartile | 2nd Quartile | 3rd Quartile | 4th Quartile |
| **sBP SD** | **Liu et al., 2020[10]** | Reference | 0.99 [0.88-1.13] | 1.02 [0.90-1.16] | 1.01 [0.89-1.14] |
| | **Gu et al., 2022[14]** | Reference | 0.80 [0.71–0.90] | 0.82 [0.73–0.92] | 0.96 [0.86–1.08] |
| **dBP SD** | **Liu et al., 2020[10]** | Reference | 1.04 [0.92-1.19] | 1.15 [1.02-1.30] | 1.11 [0.98-1.25] |
| | **Gu et al., 2022[14]** | Reference | 0.80 [0.71–0.90] | 0.79 [0.70–0.89] | 0.95 [0.85–1.07] |
| **sBP CV** | **Liu et al., 2020[10]** | Reference | 1.03 [0.91-1.16] | 1.03 [0.91-1.17] | 1.00 [0.89-1.13] |
| | **Gu et al., 2022[14]** | Reference | 0.80 [0.71–0.90] | 0.88 [0.78–0.99] | 0.97 [0.86–1.09] |
| **dBP CV** | **Liu et al., 2020[10]** | Reference | 1.14 [1.01-1.29] | 1.10 [0.97-1.25] | 1.16 [1.03-1.31] |
| | **Gu et al., 2022[14]** | Reference | 0.85 [0.76–0.96] | 0.91 [0.81–1.03] | 0.95 [0.84–1.08] |
| **SGA** | | 1st Quartile | 2nd Quartile | 3rd Quartile | 4th Quartile |
| **sBP SD** | **Liu et al., 2020[10]** | Reference | 1.03 [0.94-1.12] | 1.08 [0.98–1.18] | 1.12 [1.03–1.23] |
| | **Gu et al., 2022[14]** | Reference | 1.25 [1.07–1.46] | 1.07 [0.91–1.25] | 1.35 [1.16–1.58] |
| **dBP SD** | **Liu et al., 2020[10]** | Reference | 0.96 [0.88–1.06] | 1.06 [0.96–1.16] | 1.15 [1.06–1.26] |
| | **Gu et al., 2022[14]** | Reference | 1.00 [0.85–1.18] | 1.09 [0.93–1.28] | 1.32 [1.14–1.54] |
| **sBP CV** | **Liu et al., 2020[10]** | Reference | 1.04 [0.95–1.14] | 1.07 [0.98–1.17] | 1.12 [1.02–1.23] |
| | **Gu et al., 2022[14]** | Reference | 1.12 [0.96–1.31] | 1.20 [1.02–1.40] | 1.26 [1.08–1.47] |
| **dBP CV** | **Liu et al., 2020[10]** | Reference | 0.94 [0.86–1.03] | 1.04 [0.95–1.14] | 1.14 [1.05–1.25] |

| | | | | | |
|---|---|---|---|---|---|
| | **Gu et al., 2022[14]** | Reference | 1.09 [0.93–1.27] | 1.19 [1.01–1.39] | 1.38 [1.17–1.61] |
| **Fetal Distress** | | 1st Quartile | 2nd Quartile | 3rd Quartile | 4th Quartile |
| **sBP SD** | **Gu et al., 2022[14]** | Reference | 1.00 [0.94–1.07] | 1.04 [0.97-1.11] | 1.05 [0.98-1.12] |
| **dBP SD** | **Gu et al., 2022[14]** | Reference | 1.09 [1.02–1.16] | 1.17 [1.10–1.25] | 1.19 [1.11–1.27] |
| **sBP CV** | **Gu et al., 2022[14]** | Reference | 1.02 [0.95–1.08] | 1.05 [0.98-1.12] | 1.05 [0.98-1.12] |
| **dBP CV** | **Gu et al., 2022[14]** | Reference | 1.05 [0.98–1.12] | 1.13 [1.05–1.20] | 1.22 [1.14–1.30] |
| **5-Minute Apgar Score** | | 1st Quartile | 2nd Quartile | 3rd Quartile | 4th Quartile |
| **sBP SD** | **Gu et al., 2022[14]** | Reference | 0.88 [0.46–1.66] | 0.66 [0.33–1.32] | 0.66 [0.33–1.31] |
| **dBP SD** | **Gu et al., 2022[14]** | Reference | 0.49 [0.23–1.05] | 0.85 [0.45–1.62] | 0.84 [0.44–1.62] |
| **sBP CV** | **Gu et al., 2022[14]** | Reference | 0.73 [0.38–1.40] | 0.69 [0.36–1.34] | 0.57 [0.28–1.14] |
| **dBP CV** | **Gu et al., 2022[14]** | Reference | 0.67 [0.32–1.41] | 1.18 [0.61–2.26] | 0.80 [0.39–1.66] |
| | **LBW** | 1st Quartile | 2nd Quartile | 3rd Quartile | 4th Quartile |
| **sBP SD** | **Liu et al., 2020[10]** | Reference | 1.13 [0.96–1.34] | 1.16 [0.97–1.37] | 1.31 [1.11–1.53] |

| | | | | | |
|---|---|---|---|---|---|
| **dBP SD** | **Liu et al., 2020[10]** | Reference | 0.97 [0.82–1.16] | 1.22 [1.04–1.44] | 1.28 [1.09–1.50] |
| **sBP CV** | **Liu et al., 2020[10]** | Reference | 1.13 [0.96–1.34] | 1.18 [1.00–1.40] | 1.22 [1.04–1.44] |
| **dBP CV** | **Liu et al., 2020[10]** | Reference | 1.14 [0.96–1.34] | 1.13 [0.96–1.34] | 1.39 [1.18–1.63] |

CV indicates coefficient of variation; dBP, diastolic blood pressure; LBW, low birth weight; PTB, preterm birth; SD, standard deviation; sBP, systolic blood pressure, SGA, small-for-gestational-age.

ORs for association between adverse perinatal outcomes and measures of BP variability in all participants. BP variability used in four quartiles.
